# Leveraging beneficial microbiome-immune interactions via probiotic use in cancer immunotherapy

**DOI:** 10.3389/fimmu.2025.1713382

**Published:** 2025-12-15

**Authors:** Chinmay T. Jani, Kyle Edwards, Charmi Bhanushali, Xena Zheng, Ana S. Salazar, Gilberto Lopes, Dionysios C. Watson

**Affiliations:** 1 Division of Medical Oncology, Department of Medicine, Sylvester Comprehensive Cancer Center, University of Miami, Miami, FL, United States; 2 Department of Internal Medicine, St Vincent Medical Center, Worcester, MA, United States

**Keywords:** tumor micreoenvironment (TME), immune check inhibitor (ICI), probiotics, gut microbiome, immunotherapy, cancer

## Abstract

The gut microbiome is a critical regulator of systemic immunity and a major modulator of response to cancer immunotherapy with immune checkpoint inhibitors (ICIs). However, the clinical implementation of microbiome-inspired therapies that leverage these associations have proven challenging. Probiotics—live microorganisms thought to confer health benefits as part of food or food supplements—have gained increasing attention as readily testable, low-toxicity agents with potential of favorably influencing host–microbiome–immune interactions in the context of cancer immunotherapy. In this review, we critically evaluate the growing body of evidence supporting the role of probiotics in enhancing ICI efficacy and summarize published and ongoing clinical trials formally testing their role as adjuncts to cancer immunotherapy. Probiotics have been shown in preclinical murine models to exert immunomodulatory effects, including activation and maturation of dendritic cells, enhancement of MHC-I-mediated antigen presentation, modulation of cytokine profiles, and promotion of pro-inflammatory macrophage polarization. Probiotics also regulate adaptive immunity via microbial metabolites such as short-chain fatty acids (SCFAs), inosine, and tryptophan derivatives that support effector T cell activation and reduce T cell exhaustion. Cross-reactivity between microbial and tumor-associated antigens (molecular mimicry) further underscores the potential of probiotic strains to stimulate antitumor responses. In these models, supplementation with specific bacterial strains such as *Bifidobacterium* spp., *Lactobacillus* spp., *Clostridium butyricum*, and *Akkermansia muciniphila* enhanced ICI responses across tumor types including melanoma, lung cancer, and colorectal cancer. These findings are in part supported by early-phase clinical studies and retrospective cohorts, particularly in lung and renal cancers, where probiotic use has been associated with improved progression-free and overall survival. However, most clinical data are observational, and the field lacks standardized probiotic formulations and dosing protocols. To transition probiotics from food supplements to clinically validated immunotherapy adjuncts, rigorous mechanistic, translational, and clinical studies are necessary. These approaches have the potential to define mechanism-of-action, identify immunologically active strains, and inform rational clinical trial design. With careful development, probiotics hold promise as cost-effective, scalable, and personalized tools to optimize the efficacy and safety of cancer immunotherapy.

## 1 Introduction

Immunotherapies, particularly immune checkpoint inhibitors (ICIs), have broadly revolutionized cancer care by enhancing the immune system’s ability to recognize and destroy cancer cells (1, 2). ICIs target inhibitory pathways such as CTLA-4 and PD-1/PD-L1 that tumors exploit to suppress T-cell activity, thereby restoring antitumor immune responses (3, 4). Their clinical success has transformed the treatment landscape across multiple malignancies, including melanoma, non-small cell lung cancer (NSCLC), renal cel carcinoma (RCC), and urothelial carcinoma (5–7). However, a significant proportion of patients do not benefit from ICIs. Resistance, either primary (no initial response) or acquired (progression after initial benefit) limits long-term efficacy (8).

Resistance mechanisms include both tumor-intrinsic factors, such as loss of antigen presentation, abberations in interferon signaling, and upregulation of alternate checkpoints (LAG-3, TIM-3, TIGIT); and tumor-extrinsic factors, such as infiltration by regulatory T cells (Tregs), myeloid-derived suppressor cells (MDSCs), and a broadly immunosuppressive tumor microenvironment (TME) (9–12).

One of the most compelling emerging areas in immunotherapy research involves the microbiome’s influence on both ICI efficacy and toxicity. Distinct microbial ecosystems are present across multiple mucosal and epithelial surfaces - including the skin, lungs, and vaginal mucosa (13). These microbiomes each contribute to site-specific immune regulation - shaping pulmonary immune tone, maintaining epithelial homeostasis, and preserving mucosal barrier integrity. Collectively, these microbial communities influence local inflammation and may indirectly modulate systemic and antitumor immune responses (14, 15). Despite this diversity, most mechanistic and clinical investigations to date have centered on the gut microbiome, owing to its dense microbial load, metabolic versatility, and evidence of profound bidirectional interactions with the host immune system.

The gut microbiome, which is often considered the “second genome” of humans, consists of trillions of microorganisms in the gastrointestinal tract (16). It plays a central role in regulating systemic immunity and shaping responses to cancer immunotherapy (17–19). It influences cytokine signaling, antigen presentation, and effector T-cell function (19, 20). Notably, disruptions in microbial diversity such as those caused by antibiotics or dysbiosis, defined as a disturbance of gut microbiota homeostasis due to imbalances in microbial composition, function, or distribution, are linked to poorer ICI responses (21). In contrast, enrichment of specific bacterial species including *Akkermansia muciniphila*, *Clostridium butyricum*, *Bifidobacterium* spp., *Faecalibacterium* spp., and others has been associated with enhanced antitumor immunity and improved clinical outcomes (22–26). Bacteria that preserve intestinal mucosal integrity appear to be particularly favorable in this context (27, 28). While the exact molecular mechanisms remain under investigation, current translational strategies aim to leverage this potential through microbiome-targeted interventions, including: Probiotics (live beneficial bacteria), Prebiotics (dietary substrates promoting growth of favorable microbes), Postbiotics (microbial metabolic products associated with clinical benefits), and Fecal microbiota transplantation (FMT) from ICI responders or healthy donors (27, 29). Diet is another critical factor that can modulate the gut microbiome and thereby influence immunotherapy outcomes. Numerous studies have shown that dietary patterns, especially high fiber intake are associated with enrichment of beneficial microbial taxa and improved progression-free survival in patients treated with immune checkpoint inhibitors. Notably, the DIET study showed an increase in ORR to 77% for patients receiving High Fiber Diet Intervention as compared to control group of patients (30). Other dietary interventions such as ketogenic diets and supplementation with prebiotics like inulin and polyphenols (e.g., castalagin from camu camu berry) have shown preclinical promise in modulating the microbiome to enhance ICI response; however, the clinical utility of these additional diets remains to be determined (31).

This review first summarizes current knowledge of the gut microbiome’s influence on immunotherapy and then examines the emerging evidence for probiotics as potential modulators of ICI efficacy. By synthesizing insights from mechanistic, translational, and clinical research, we highlight how probiotics may evolve from health supplements to validated adjuncts in cancer immunotherapy, provided they undergo rigorous clinical validation in the coming years.

## 2 Gut microbiome and cancer immunity

The human gut harbors a complex and dense microbial ecosystem composed of bacteria, viruses, fungi, and other microorganisms (32). While historically recognized for its role in digestion, metabolic regulation, and maintenance of gut barrier integrity, recent research has highlighted its critical involvement in shaping immune system development and function (33–35). Healthy microbial diversity is often associated with resilience against pathogens, reduced inflammation, and robust immune function. Conversely, dysbiosis, an imbalance in microbial composition, has been linked to a range of immune-related disorders, including autoimmune diseases, allergies, metabolic conditions, and carcinogenesis (34, 36–38).

### 2.1 Immunologic role of the gut microbiome

The gut microbiome exerts its influence on both local and systemic immune responses through a sophisticated interplay shaped by various environmental factors (33, 34, 39). The gastrointestinal tract is lined with gut-associated lymphoid tissue (GALT), comprising a vast network of immune cells primed to respond to microbes. Unlike peripheral lymph nodes which are most of the time sterile and quiescent, GALT is constantly exposed to foreign antigens from both commensal microbiota and infectious pathogens (40). Mucosal addressin cell adhesion molecule-1 (MAdCAM-1) is a key endothelial adhesion molecule that directs lymphocyte trafficking into the gut by binding to the α4β7 integrin on T cells (41). Its expression on high endothelial venules is critical for Treg homing to the intestinal mucosa, maintaining immune tolerance and mucosal immune balance (41). Gut dysbiosis or antibiotic-induced downregulation of MAdCAM-1 increases systemic migration of Tregs, driving their accumulation in the TME and impairing responses to immune checkpoint blockade (42, 43). Restoration of gut MAdCAM-1, either by microbial or molecular intervention, preserves local Treg populations, reduces tumor infiltration, and enhances the efficacy of immune checkpoint inhibitors in preclinical models (43).

Through interactions among microorganisms themselves and between microorganisms and host immune cells, numerous metabolites and cellular components are produced that regulate immunity at both the local and systemic levels. At the local level, the microbiome plays a crucial role in maintaining the gut epithelial barrier by promoting mucus production, enhancing tight junction integrity, and preventing pathogenic bacterial overgrowth (33, 34, 39). It also influences systemic immunity through key metabolites, short chain fatty acids (SCFAs), tryptophan derivatives, and bile acids, which regulate both innate and adaptive immune responses (34, 44, 45). They have been shown to support gut barrier integrity and exert anti-inflammatory effects (33, 34, 39). Additionally, microbial structural components known as microorganism-associated molecular patterns (MAMPs), such as lipopolysaccharide (LPS), formyl peptides, and peptidoglycan, engage host pattern recognition receptors (PRRs), initiating immune signaling cascades that contribute to both immune activation and immune regulation (34). Beyond the local effects in the gut, microbial metabolites exert systemic immunomodulatory effects through interactions in the GALT which supports the diversification, propagation, and possibly selection of systemic B cells (40).

### 2.2 Microbial metabolites and immune signaling pathways

SCFAs, such as acetate, propionate, and butyrate, are produced via bacterial fermentation of complex carbohydrates (e.g. dietary fiber) in the colon. These metabolites bind to G protein-coupled receptors (GPCRs) including GPR41, GPR43, and GPR109A, expressed on epithelial and immune cells (45). Activation of these receptors triggers intracellular signaling cascades that regulate immune cell activation, cytokine secretion, and inflammatory responses in the colon. SCFA-GPCR signaling supports Treg expansion while suppressing the differentiation of pro-inflammatory Th17 cells within the intestinal immune system (46). Beyond receptor-mediated signaling, SCFAs, particularly butyrate, also inhibit histone deacetylase (HDAC) activity, leading to increased histone acetylation, chromatin relaxation, and altered gene transcription (47–49). These epigenetic modifications further promote Treg differentiation through upregulation of FoxP3, a master transcription factor for Tregs (45, 50). In murine melanoma and pancreatic cancer models, microbial SCFAs and butyrate through HDAC inhibition, enhance the anti-tumor activity of CD8+ cytotoxic T lymphocytes leading to increased effector cytokine production and proliferation (51).

Tryptophan metabolites also play a central role in immune regulation, primarily through activation of the aryl hydrocarbon receptor (AhR) on the dendritic or T cells of the GALT. AhR signaling enhances the differentiation and proliferation of Tregs while simultaneously inhibiting pro-inflammatory Th17 cell development (52). In addition, AhR activation in dendritic cells promotes a tolerogenic phenotype, limiting their capacity to initiate inflammatory responses and instead supporting Treg expansion. Tryptophan metabolites also modulate cytokine production, downregulating IL-6, TNF-α, and IL-17, and upregulating IL-10 and TGF-β, thereby reinforcing anti-inflammatory immune responses (52–54).

Bile acids further exemplify the immunoregulatory capacity of microbial metabolites. Primary bile acids, synthesized from cholesterol in the liver, are transformed by gut microbiota into secondary bile acids (53). These metabolites bind to nuclear and membrane receptors, including Farnesoid X Receptor (FXR), Vitamin D Receptor (VDR) and Retinoid-Related Orphan Receptor gamma t (RORγt) in the lamina propria and Peyer’s patches, and G protein-coupled bile acid receptor 1 (GPBAR1 or TGR5) on gut macrophages (53, 55). Activation of FXR and VDR leads to suppression of pro-inflammatory cytokines and inhibition of NF-κB signaling. Simultaneously, inhibition of RORγt reduces Th17 cell differentiation, while stimulation of GPBAR1 promotes a shift in macrophage polarization toward an anti-inflammatory phenotype characterized by decreased expression of TNF-α, IFN-γ, IL-1β, IL-6, and CCL2 (55, 56). In murine colitis models, activation of GPBAR1 not only reprogrammed intestinal macrophages but also reduced the recruitment of circulating monocytes from the blood into the gut, demonstrating a direct systemic immune effect alongside local mucosal regulation (55).

**Figure 1:**
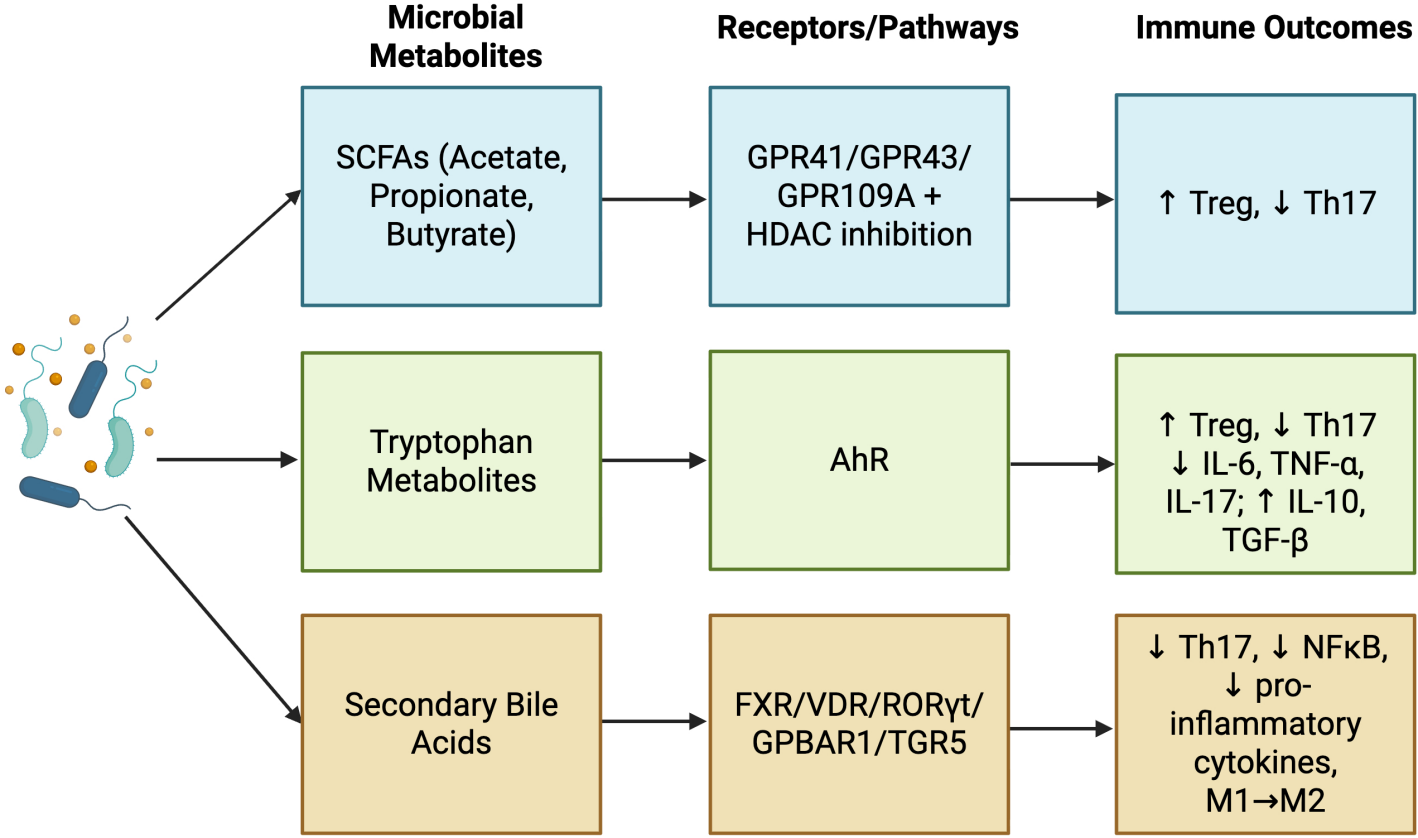
Examples of microbial metabolites and their effect on the immune responses. Short-chain fatty acids (SCFAs), tryptophan metabolites, and secondary bile acids represent some bioactive postbiotics or metabolites that shape immune responses through receptor-mediated and epigenetic mechanisms. SCFAs act via GPR41/43/109A and HDAC inhibition, tryptophan metabolites signal through AhR, and bile acids modulate FXR, VDR, RORγt, and GPBAR1/TGR5. These pathways have been shown to promote Treg differentiation, suppress Th17 responses, alter cytokine production, inhibit NF-κB signaling, and shift macrophages toward an anti-inflammatory phenotype (45–55). Created using Biorender.

Together, these pathways demonstrate that microbial metabolites influence the immune landscape through both receptor-mediated signaling and epigenetic regulation. By modulating the balance between pro- and anti-inflammatory cell populations and altering gene expression, the gut microbiome exerts both direct local effects and, via immune and cytokine trafficking, potentially far-reaching influence on systemic immunity and tumor sites. (**Figure 1**)

### 2.3 Microbiome influence on cancer immunotherapy

Over the past decade, a growing body of evidence has underscored the critical role of the gut microbiome in modulating the efficacy of ICIs (57–61). Notably, differences in gut microbial composition have been observed between patients who respond to ICIs and those who do not. Early studies reported that greater baseline gut microbiota richness and diversity were associated with improved responses to ICIs, including anti-CTLA-4 and anti-PD-1 therapies in melanoma and epithelial cancers, while antibiotic use was linked to diminished clinical benefit (58–61).

**Figure 2:**
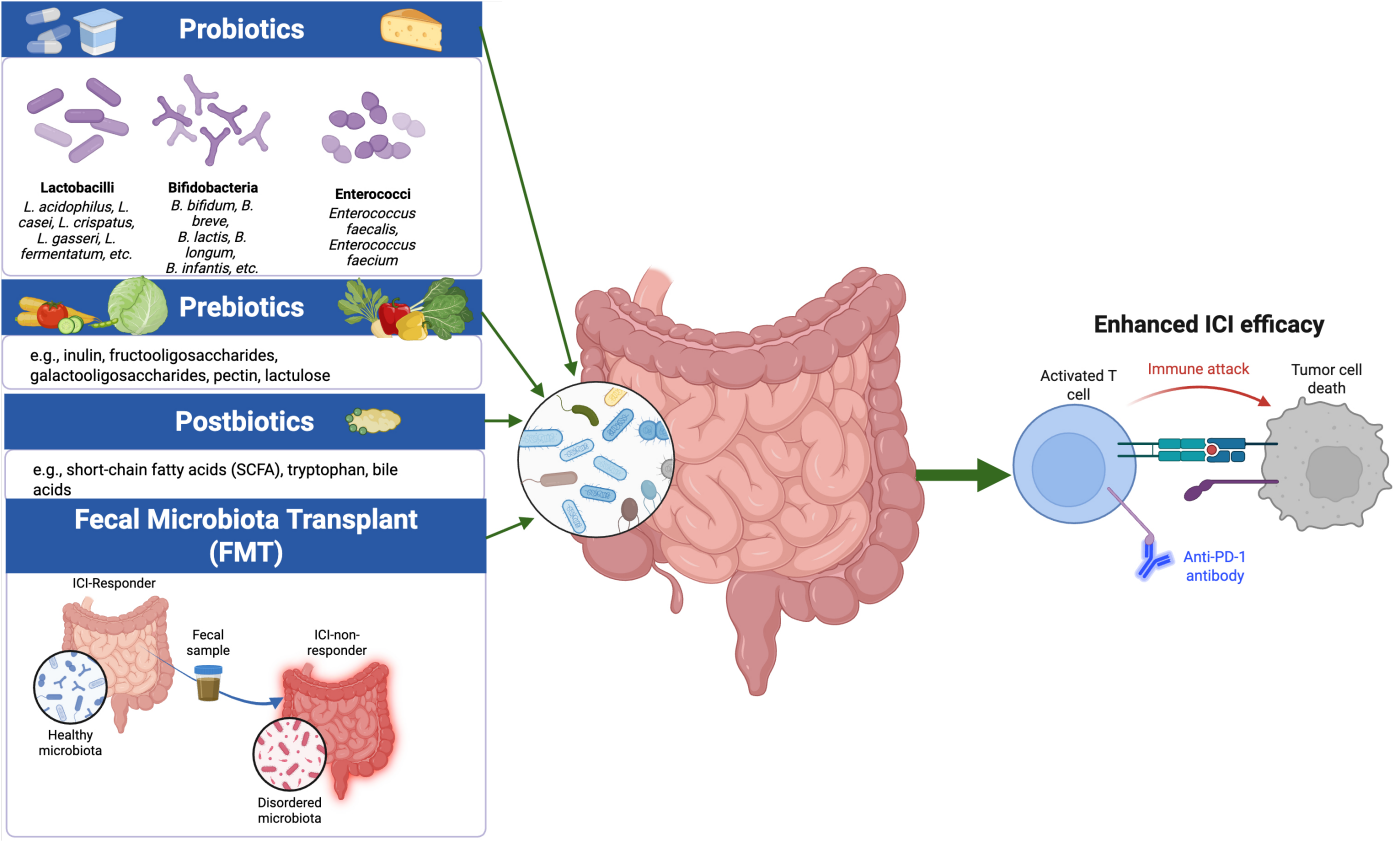
Probiotics, Prebiotics, Postbiotics, and FMT as Microbiome-Directed Strategies to Enhance ICI Efficacy Probiotics (e.g., Lactobacillus, Bifidobacterium), prebiotics (e.g., inulin, fructooligosaccharides), postbiotics (e.g., SCFAs, tryptophan, bile acids), and fecal microbiota transplantation (FMT) can increase beneficial taxa and metabolites, restore microbial diversity, and ultimately enhance antitumor T cell responses to PD-1 blockade (57–61). Created using Biorender.

Further research has identified specific microbial taxa associated with favorable treatment outcomes, including bacteria from the *Ruminococcaceae* family, *Akkermansia mucinophila*, *Alistipes indistinctus*, *Bifidobacterium longum*, and *Enterococcus faecium* (58–61). Conversely, the presence of certain bacteria or an overall loss of microbial diversity (dysbiosis) has been linked to poor therapeutic responses (59, 60). For example, *Enterocloster* spp can modulate the migration of immunosuppressive T cell subsets into tumors via downregulation of MAdCAM-1 where they suppress anti-tumor immunity and promote resistance to immunotherapy (43). These findings underscore the promise of microbiome-targeted strategies to enhance cancer immunotherapy. Interventions such as probiotics, prebiotics, postbiotics, and FMT (**Figure 2**) are gaining traction for their potential to modulate the gut microbiome and improve treatment outcomes in cancer.

How to most effectively leverage the growing understanding of the microbiome’s effect on immunity in the design of therapeutics remains a subject of intense research. Several strategies are being explored, ranging from administering live beneficial bacteria (probiotics) and dietary components that selectively nourish them (prebiotics), to delivering bioactive microbial metabolites (postbiotics) and performing fecal microbiota transplantation (FMT) using material from ICI responders or healthy donors. The Food and Agriculture Organization (FAO) and the World Health Organization (WHO) define probiotics as “live microorganisms which, when administered in adequate amounts, confer a health benefit on the host.” (62) These beneficial microbes are sometimes also delivered through fermented foods like yogurt and cheese, as well as fermented fruits, and vegetables (63, 64). Frequently used probiotic strains include *Bifidobacterium* spp. and *Lactobacillus* spp., although other Gram-positive genera such as Streptococcus, Bacillus, Enterococcus, and various yeasts are also utilized (65). Besides treatment with live bacteria to alter the microbiome, other studies focus on supplementing dietary content of molecules that are metabolized by microbes (prebiotics) to produce downstream metabolites previously linked to a desired effects. Recent studies have demonstrated the role of inulin, a naturally occurring prebiotic fiber that can reinforce cancer immunosurveillance by activating γT cells, which play a crucial role in the anti-tumor immune response (66). Furthermore, inulin supplementation has been associated with increased production of SCFAs by microbes in the gut, which have in turn been shown to promote a favorable immune environment and improve systemic immune responses (67). Inulin supplementation was shown to inhibit tumor growth in melanoma and colorectal cancer mouse models by shifting gut microbiota taxa towards those that increase anti-tumor immune activity (68). Additionally, inulin has been shown to enhance the effects of various cytotoxic drugs. It can modulate the microbiota, resulting in increased fecal SCFA levels and improved systemic antitumor immunity in mouse models treated with anti-PD-1 antibodies (69, 70). In contrast, postbiotics are non-viable microbial cells or their components including structural fragments, peptides, and metabolites (SCFA, tryptophan, Bile acids) that can exert health benefits despite lacking viability. The International Scientific Association for Probiotics and Prebiotics (ISAPP) defines postbiotics as “a preparation of inanimate microorganisms and/or their components that confer a health benefit on the host.” (63, 65)

FMT, which involves transferring fecal material from healthy or ICI-responsive donors to patients, offers a comprehensive approach to restoring gut microbial diversity and function. Preclinical studies have shown that FMT from ICI responders enhances antitumor efficacy in germ-free mice and non-responder patients (59, 60, 71). Gopalakrishnan et al. reported improved outcomes in mice receiving FMT from melanoma responders, whereas transplants from non-responders failed to confer benefit (59). Similarly, Sivan et al. demonstrated that oral administration of *Bifidobacterium* spp. in melanoma and bladder cancer murine models improved tumor control by enhancing dendritic cell function and CD8+ T cell priming and infiltration in the tumor microenvironment (71). Early-phase clinical trials by Baruch, Davar, Routy and Kim have shown that FMT followed by anti–PD-1 therapy can overcome resistance in refractory melanoma, with sustained microbial changes and clinical benefit (20, 61, 72, 73).

Among these microbiome-targeted strategies, probiotics warrant particular attention because they represent a practical, scalable, and non-invasive approach to modulating gut microbial composition. Probiotics can be standardized, administered orally, and integrated into routine clinical care with relative ease.

## 3 Probiotics as ICI therapy adjuncts

An expanding body of research highlights the capacity of probiotics to modulate host immune responses and enhance the efficacy of cancer immunotherapy. These live microorganisms, when administered in adequate amounts, can influence both systemic and local immune pathways, with emerging data supporting their role as adjuncts to ICIs. Preclinical and clinical studies suggest that specific probiotic strains can shape the TME, promote effector T cell function, and modulate the gut microbiota to favor antitumor immunity.

Growing clinical evidence highlights the role of microorganisms in both promoting and preventing cancer. Certain microbes, such as *Helicobacter pylori*, are known to contribute to carcinogenesis, for example in gastric cancer (74). In contrast, various probiotic strains have demonstrated protective effects. Administered as a probiotic in preclinical models, *Lactobacillus rhamnosus GG* has been shown to promote apoptosis in colon cancer cells, while findings from the EPIC Italy cohort study revealed that yogurt consumption rich in *Streptococcus thermophilus* and *Lactobacillus delbrueckii subsp. bulgaricus* was inversely associated with colon cancer risk (74, 75). *L. rhamnosus LC705* and *Propionibacterium freudenreichii* co-administered as a probiotic supplement in human intervention studies was found to limit aflatoxin absorption and may help reduce liver cancer risk (76). Similarly, *Lactobacillus casei* strain Shirota has also been associated with enhanced macrophage and T-cell activity, potentially reducing the recurrence of bladder and colorectal cancers (77, 78).

Beyond prevention, probiotics have shown clinical promise in enhancing treatment efficacy. Probiotic supplementation has been associated with improved gut microbiota composition that resemble that of healthy individuals, reduction in species such as *Fusobacterium* and *Peptostreptococcus*, which are commonly isolated in cancer patients with poor outcomes, and strengthened intestinal barrier function (79). In colorectal cancer, several clinical studies have demonstrated that probiotics can augment chemotherapy response. For instance, probiotic strains with *Lactobacillus acidophilus* and *L. casei* increased apoptosis in colorectal cancer cells treated with 5-fluorouracil, even in resistant cell lines (79). *L. plantarum* enhanced 5-fluorouracil sensitivity by disrupting the Wnt/β-catenin signaling pathway (79). In the perioperative setting, probiotic administration in colorectal cancer surgery led to reduced bacterial translocation, preserved mucosal integrity, and improved postoperative recovery (80). Additionally, a retrospective study found that pancreatic cancer patients receiving probiotics, regardless of the type alongside palliative chemotherapy may experience improved overall survival (OS) (12 vs 10 months, p = 0.026) (81).

A critical application of probiotics in oncology involves mitigating treatment-related toxicities. In lung cancer patients receiving chemotherapy, *Clostridium butyricum* administration was associated with decreased incidence of diarrhea and modulation of systemic inflammation (82). A systematic review of eight randomized clinical trials found that probiotic supplements which utilized strains such as *Lactobacillus* spp. and *Bifidobacterium* spp. were associated with decreased TNF levels and improved quality of life in breast cancer survivors with lymphedema (74). Another umbrella meta-analysis found that probiotics decreased infection rates, oral mucositis, and overall treatment-related complications in patients with malignancies (83).

Although colon cancer cell models have been widely used to study the antiproliferative effects of probiotics, they inadequately capture the complex host–microbiome–immune interactions central to probiotic efficacy in immuno-oncology. Notably, Spencer et al. reported that non-standardized, commercially available *Lactobacillus* spp. and *Bifidobacterium* spp. probiotic supplements were associated with impaired ICI response and reduced interferon-γ–positive cytotoxic T cells in both preclinical models and melanoma patients, suggesting potential detrimental effects during checkpoint blockade (84). Importantly, it analyzed consumer-grade supplements, rather than pharmaceutical or characterized single strains, both in preclinical (murine) models and a large prospective human cohort (84). In contrast, a meta-analysis integrating eight retrospective and four prospective studies including the Spencer et al. cohort found a positive association between probiotic use, OS, and ORR among patients receiving ICIs (85). However, limited data prevented subgroup analyses by probiotic type, duration, ICI class, or cancer type, leading to heterogeneity in outcomes. Collectively, these divergent findings highlight the need for well-designed, randomized trials with strain-level resolution to clarify the conditions under which probiotics may serve as safe and effective adjuncts to ICI therapy (85).

### 3.1 Mechanisms of action

Probiotics can modulate both innate and adaptive immune responses, notably by enhancing dendritic cell (DC) function, antigen presentation, cytokine production, and T cell activation within the tumor microenvironment. A key mechanism involves probiotic-induced activation and maturation of DCs, which improves MHC-I-mediated antigen presentation in lymphoid tissues like Peyer’s patches and lymph nodes, thereby increasing tumor-specific CD8+ T cells (71, 86–88). In a murine melanoma model, oral *Bifidobacterium* spp. administration led to expanded T cell populations in tumor-draining lymph nodes and increased IFN-γ production, likely driven by DC activation. This was accompanied by upregulation of genes involved in MHC-I presentation (Tapbp), DC maturation (Relb), chemokine signaling (Cxcl9), and type I interferon pathways (Irf1), promoting robust CD8+ T cell-mediated antitumor responses in the TME (71). Supporting this, another study identified 11 bacterial strains that enhanced T cell activation via DC–MHC I interaction (89).

**Figure 3:**
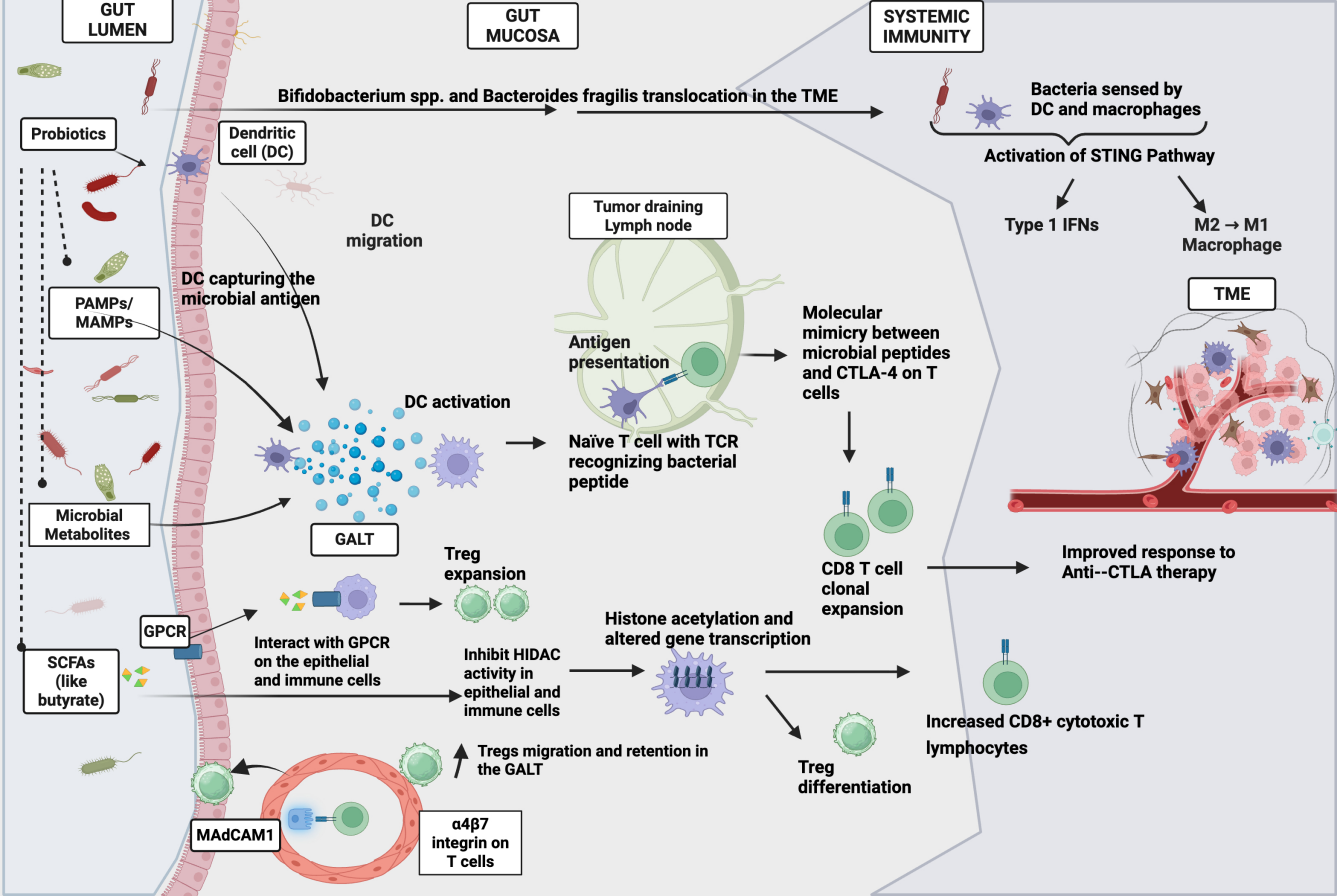
Potential mechanisms by which probiotics enhance antitumor immunity and improve ICI responses. This schematic summarizes key and emerging pathways by which probiotics and their metabolites may modulate host immunity and augment the efficacy of immune checkpoint inhibitors (ICIs), as described in current preclinical and translational literature: Probiotics can influence both local and systemic immune responses through multiple mechanisms. In the gut, probiotics and commensal bacteria produce short-chain fatty acids (SCFAs) such as butyrate, which acts through G-protein-coupled receptors (GPCRs) on epithelial and immune cells, supporting regulatory T cell (Treg) expansion and suppressing pro-inflammatory Th17 cells. Butyrate and other SCFAs also act as histone deacetylase (HDAC) inhibitors, leading to increased histone acetylation at gene promoters (e.g., SOCS1, FoxP3), chromatin relaxation, and improved gene transcription favoring antitumor T cell differentiation and immune tolerance (45–53, 106). Pattern-recognition of microbial molecules (PAMPs/MAMPs) by dendritic cells (DCs) and epithelial cells in the gut initiates DC activation and migration from the gut-associated lymphoid tissue (GALT) to tumor-draining lymph nodes (TDLNs). There, enhanced antigen presentation by DCs promotes naive T cell priming and expansion, including cross-priming via molecular mimicry between microbial and tumor antigens (89–91, 97, 101, 102). Probiotic bacteria and their metabolites may also translocate to the tumor microenvironment (TME), where they are sensed by infiltrating DCs and macrophages, activating the STING pathway and inducing Type I interferons (Type 1 IFNs). This signaling polarizes tumor-associated macrophages from an immunosuppressive M2 phenotype toward the antitumor M1 phenotype, further enhancing CD8+ T cell recruitment and activity in the TME (92–96). Created using Biorender.

Probiotics also regulate cytokine production. Commensal bacteria interact with DCs and epithelial cells in the gut through pattern recognition receptors, leading to the release of cytokines such as TNF-α, TGF-β, IL-12, and IL-10 locally (90). This gut-derived cytokine milieu induces maturation and activation of innate and adaptive immune cells, which can lead to systemic effects, including priming and recruitment of effector T cells in secondary lymphoid organs and ultimately the tumor microenvironment in murine models (86, 90, 91) Macrophage polarization represents another important axis of probiotic-driven immune modulation. Tumor-associated macrophages typically exhibit immunosuppressive (“M2-like”) polarization. Orally administered *Bifidobacterium* spp. and *Bacteroides* fragilis in mouse colon adenocarcinoma models, were showed to accumulate in the TME using bacterial tracing and cultures (92, 93). Furthermore, in the EG7 lymphoma mode, Bifidobacterium selectively accumulated within the hypoxic regions of the TME following systemic administration (93). Within the tumor, these bacteria are sensed by infiltrating macrophages and dendritic cells, which activate the STING pathway in response to bacterial DNA and signals. This activation triggers type I interferon production and polarizes macrophages toward the pro-inflammatory, antitumor (M1-like) phenotype (93–96). (**Figure 3**)

Probiotics also shape adaptive immunity through multiple mechanisms. Microbial-derived pathogen- and microbe-associated molecular patterns (PAMPs/MAMPs) can cross the gut mucosal barrier, reach lymphoid organs or tumor sites, and prime T cells for antitumor responses via molecular mimicry. Probiotic metabolites further drive immune activation; for instance, inosine produced by *Bifidobacterium pseudolongum* which was administered as a probiotic in murine cancer models, enhanced MHC-I expression and activates IFN-γ and TNF signaling, leading to increased CD8+ T cell proliferation and reduced PD-1 expression, thereby limiting T cell exhaustion in TME (97).

However, our understanding of how the potential effects of probiotics on the immune system translates to beneficial reprogramming of the tumor microenvironment and enhancement of ICI response remains incomplete. Most mechanistic insights are derived from murine models, which may not fully recapitulate human immune-microbiome interactions. In humans, evidence largely consists of correlative studies or small pilot trials, leaving uncertainty about causality, optimal strains, and dosing strategies. Key questions remain regarding the identification of the most effective probiotic strains, strategies to account for patient-specific microbiome variability, and the safety of administering live microorganisms in immunocompromised populations.

### 3.2 Preclinical evidence supporting probiotic–ICI synergy

Some studies have suggested that probiotic supplementation can help restore microbiome composition, preserve gut barrier integrity, and prevent systemic immunosuppression, potentially maintaining or improving response to ICI therapy (61, 98–100). It is found that while ICIs can “release the brakes” on T cells, the presence of certain commensal bacteria appears to intensify this T-cell mediated attack. Commensal bacteria act as natural adjuvants, priming innate and adaptive immunity in the gut through microbial products that activate Toll-like receptors and inflammasome pathways, enhancing DC maturation and cytokine production to support T cell activation (61),.

Similarly, *Bifidobacterium breve* and *Bifidobacterium longum* have been shown to facilitate DC activation and T-cell priming. In mouse models of melanoma, oral administration of *Bifidobacterium* spp. significantly enhanced antitumor immunity to a degree comparable to PD-L1 blockade. When combined, the two treatments nearly eradicated tumors, suggesting a synergistic effect likely mediated by increased CD8+ T cell infiltration (71). Further mechanistic insights were provided by Vetizou et al., who found that the antitumor activity of CTLA-4 inhibition depends on specific *Bacteroides* species, including *B. thetaiotaomicron* and *B. fragilis*. Oral supplementation with *B. fragilis* in murine models induced Th1 immune responses in tumor-draining lymph nodes and promoted DC maturation within tumors. Even in germ-free mice, this intervention enhanced CTLA-4 blockade efficacy via IL-12–dependent Th1 responses (101). In human pancreatic cancer, T cells were shown to recognize both bacterial antigens and homologous tumor neoantigens (102), suggesting that if matching antigens were delivered through probiotic administration, it could potentially be used to prime or boost antitumor T cell responses via molecular mimicry. In the Bacteroides–CTLA-4 model, T cell responses against B. thetaiotaomicron and B. fragilis were correlated with tumor regression (101).

Akkermansia muciniphila has also been shown to strengthen the gut lining and help DC maturation, which leads to more CD8+ T cells entering tumors (25, 61, 84, 103). Lactobacillus rhamnosus and Lactobacillus reuteri can also influence T-cell differentiation and cytokine production, potentially reversing immune exhaustion in patients resistant to ICI (84). In a melanoma mouse model, daily oral administration of Lactobacillus reuteri alongside PD-L1 and CTLA-4 inhibitors significantly improved antitumor activity and survival (104). Lactobacillus reuteri was found to reach tumors outside the gut and reshape the TME into a more immune-activating state, characterized by IFN-γ production from CD4+ Th1 and CD8+ Tc1 cells. This was accompanied with increased immune cell proliferation and upregulation of CCL5 and CCL4 in CD8+ T cells, enhancing their recruitment into the tumor. These T cells also showed higher Granzyme B expression, reflecting greater cytotoxic potential (104).

In mice with colorectal cancer, supplementation with Lacticaseibacillus rhamnosus enhanced the effectiveness of ICI. This effect was driven by increased production of SCFAs particularly butyric acid, which promoted CD8+ T cell infiltration and activity while suppressing Tregs in the TME (105). Notably, butyrate generated by Faecalibacterium prausnitzii in murine colorectal cancer models, acts as a histone deacetylase (HDAC) inhibitor, leading to acetylation of histone proteins at the suppressor of cytokine signaling 1 (SOCS1) promoter (106). This upregulates SOCS1, dampens the JAK-STAT signaling pathway, and mediates tumor-suppressive and immunomodulatory effects *in vivo* in the TME (25).

Together, these preclinical studies (**Table 1**) demonstrate that probiotics may augment ICI responses through diverse mechanisms, including improved antigen presentation, modulation of cytokine signaling, restoration of microbial homeostasis, and production of immunoregulatory metabolites. However, while murine models provide critical mechanistic insights and proof-of-concept data, their predictive value for human efficacy is limited due to differences in microbiome composition, immune architecture, and reliance on germ-free or antibiotic-treated conditions. Consequently, translating these findings into clinical benefit requires rigorous validation through human correlative studies and well-designed clinical trials.

**Table 1:** Preclinical Evidence supporting Probiotics- ICI synergy.

Title	Location	Type of study	Cancer of focus	Probiotic	Results
Faecalibacterium prausnitzii strain EXL01 boosts efficacy of immune checkpoint inhibitors.	France	Observational human + Mouse preclinical	NSCLC	Faecalibacterium prausnitzii (strain EXL01)	High baseline F. prausnitzii abundance predicts better immune checkpoint inhibitor response. Oral administration of the EXL01 strain restores ICI efficacy after antibiotic-induced microbiota disruption without altering fecal diversity.
The commensal microbiome is associated with antiPD1 efficacy in metastatic melanoma patients.	USA	Observational human study + mouse fecal microbiota transplant	Melanoma	Bifidobacterium longum, Collinsella aerofaciens, and Enterococcus faecium (beneficial Operational Taxonomic Unit- OUT)	All patients with beneficial/nonbeneficial OTU ratio > 1.5 achieved a RECIST-defined response.
Gut microbiome influences efficacy of PD-1–based immunotherapy against epithelial tumors.	France	Observational human study + mouse FMT experiment	Advanced cancers (eg- NSCLC, RCC)	Akkermansia muciniphila	OR 4.73 (95% CI: 1.79–12.5, p= 0.002).
Gut microbiome modulates response to antiPD1 immunotherapy.	USA	Observational human + Mouse FMT experiment	Metastatic melanoma	Ruminococcaceae	Responders had greater relative abundance of bacteria from the Ruminococcaceae family (p < 0.01). Fecal transplants from responders into germ-free mice led to significantly improved tumor control (log-rank P < 0.05).
Anticancer immunotherapy by CTLA-4 blockade relies on the gut microbiota.	France	Mouse preclinical study + human-microbiota FMT	Melanoma	Bacteroides fragilis, Bacteroids thetaiotaomicron	Bacteroides fragilis, thetaiotaomicron on fecal transplant into germ-free mice restored CTLA-4 blockade efficacy, reduced colitis, and showed higher fecal abundance of B. fragilis (P < 0.01) that inversely correlated with tumor size after treatment.
Dietary fiber and probiotics influence the gut microbiome and melanoma immunotherapy response.	USA	Human observational + mouse preclinical study	Melanoma	Oral probiotics (not strain-specific); dietary fiber.	For probiotic use, median PFS 17 vs. 23 months (HR = 1.30, 95% CI 0.82–2.07, p = 0.27) and response rate 59% vs. 68% (OR = 0.79, 95% CI 0.37–1.66, p = 0.52)
Dietary tryptophan metabolite released by intratumoral Lactobacillus reuteri facilitates immune checkpoint inhibitor treatment.	USA	Preclinical mouse melanoma study + human correlative data	Melanoma	Lactobacillus reuteri	Daily oral administration of Lactobacillus reuteri alongside PD-L1 and CTLA-4 inhibitors significantly improved antitumor activity and survival
Lacticaseibacillus rhamnosus Probio-M9 enhanced the antitumor response to anti-PD-1 therapy by modulating intestinal metabolites.	China	Mouse preclinical study	Colorectal cancer	Lacticaseibacillus rhamnosus	Probio-M9 intervention strengthened the anti-PD-1-based tumor inhibition (p<0.05).
Lysates of Lactobacillus acidophilus combined with CTLA-4-blocking antibodies enhance antitumor immunity in a mouse colon cancer model.	China	Mouse preclinical study	Colorectal cancer	Lactobacillus acidophilus	Significantly reduced tumor incidence (p = 0.014), improved body weight (p = 0.009), and enhanced CD8+ T cell infiltration (p < 0.05).
Commensal Bifidobacterium promotes antitumor immunity and facilitates anti-PD-L1 efficacy.	USA	Mouse preclinical study	Melanoma	Bifidobacterium spp	Tumor growth was significantly reduced and CD8+ T cell infiltration enhanced (p = 0.01) in the group with combination of Bifidobacterium spp. and αPD-L1 compared to controls treated with αPD-L1 only.
Dietary fiber and probiotics influence the gut microbiome and melanoma immunotherapy response.	USA	Preclinical and observational study	Melanoma	Commercially available probiotic supplements containing Bifidobacterium longum (probiotic 1) and Lactobacillus rhamnosus GG (probiotic 2)	Melanoma patients: HR probiotic users vs. nonusers= 1.30, 95% CI, 0.82-2.07. OR for response with probiotic use= 0.79, 95% CI, 0.37-1.66. Preclinical mouse models: mice receiving probiotics had significantly larger tumor volumes compared to controls (p=0.04 and p=0.01 for two probiotic formulations).
Post-Antibiotic Gut Mucosal Microbiome Reconstitution Is Impaired by Probiotics and Improved by Autologous FMT	USA	Preclinical mouse study	Melanoma mouse models	11-strain commercial probiotic	Alpha diversity post-antibiotic never returned to baseline in probiotics users even 5 months post-treatment, while it was restored within 1–2 days in the autologous FMT and spontaneous groups (p < 0.01 at all timepoints through day 180).

### 3.3 Probiotics-ICI synergy in clinical studies

Several bacterial strains have demonstrated immunostimulatory properties that may potentiate the effects of cancer immunotherapy. These findings have led to the development of rationally designed probiotics, combining strains with complementary immunomodulatory effects to optimize immunotherapy outcomes. Several retrospective studies from Japan between 2020 and 2022 explored the impact of various probiotics in patients with non-small cell lung cancer (NSCLC). One of the earliest studies evaluated 118 patients with advanced NSCLC, 39 of whom received the Clostridium butyricum MIYARI 588 strain before or after ICI therapy. Probiotic use was associated with increased *Bifidobacterium* spp. abundance, reduced gut epithelial damage, and prolonged median progression-free survival (PFS) (250 vs. 111 days) and median overall survival (OS) (not reached vs. 361 days), even among those who had received antibiotics (107).

In a larger retrospective cohort, Morita et al. assessed 927 patients with advanced NSCLC. Among those treated with ICI monotherapy, commercially available multi-strain and single-strain probiotic use significantly improved PFS (7.9 vs. 2.9 months) and OS (not attained vs. 13.1 months). In patients receiving ICIs combined with chemotherapy, probiotics improved OS (not attained vs. 22.6 months) but had no significant effect on PFS (8.8 vs. 8.6 months) (108). Similarly, Takada et al. al analyzed 294 NSCLC patients receiving PD-1 inhibitors and found that probiotics (including *Bifidobacterium* spp., C. butyricum, and antibiotic-resistant lactic acid bacteria) were associated with improved disease control and longer PFS (HR 1.73 [1.42–2.11]), although OS was not significantly affected (HR 1.40 [1.13–1.74]). Subgroup analyses suggested greater benefit in never-smokers and patients with poor ECOG performance status (109).

One of the few prospective studies evaluating probiotics in lung cancer included 253 patients with advanced disease, of whom 71 received commercially available multi-strain probiotic formulations, most often including *Lactobacillus acidophilus, Bifidobacterium lactis, Streptococcus thermophilus, Bacillus subtilis, Enterococcus faecium, Lactobacillus rhamnosus*, and *Bacillus licheniformi*. While no significant difference in median progression-free survival (PFS) was seen among NSCLC patients (16.5 vs. 12.3 months, p = 0.56), patients with small cell lung cancer (SCLC) showed significantly improved PFS with probiotic use (11.1 vs. 7.0 months, p = 0.049), indicating potential histology-specific benefits) (110). This discrepancy from prior studies may reflect differences in patient baseline gut microbiome diversity, probiotic strain and dosing heterogeneity, or the influence of cancer histotype on microbiome-immune reaction. Another prospective study from China assessed the impact of antibiotics and probiotics on immunotherapy outcomes in patients with advanced EGFR-mutant NSCLC. Probiotic use did not influence PFS, but antibiotic use was associated with shorter PFS, suggesting that gut microbiome disruption from antibiotics may impair clinical benefit from immunotherapy. Additionally, responders to immune checkpoint inhibitors exhibited distinct metabolic profiles, with higher levels of deoxycholic acid, glycerol, and quinolinic acid, whereas non-responders had elevated L-citrulline (111).

Beyond lung cancer, an open-label single-center study by Dizman et al. assessed Clostridium butyricum (CBM588) in treatment-naïve metastatic renal cell carcinoma (mRCC) patients. Those receiving nivolumab–ipilimumab plus CBM588 had significantly improved PFS (12.7 vs. 2.5 months) and a numerically higher ORR (58% vs. 20%) (112). In a follow-up study of treatment-naïve mRCC patients receiving cabozantinib and nivolumab, the CBM588 group again showed superior ORR (74% vs. 20%, p = 0.01) and higher 6-month PFS (84% vs. 60%), indicating enhanced clinical activity (113). However, no differences in *Bifidobacterium* spp. abundance or alpha diversity were observed between arms, which suggests that while probiotic supplementation appears to enhance ICI efficacy, the absence of consistent biomarker changes means that the underlying mechanisms remain poorly defined. In a retrospective study of 352 patients with advanced digestive tract cancers (hepatocellular, colorectal, and gastric cancers) those treated with PD-1 inhibitors plus antiangiogenic agents and probiotics had a higher ORR (OR 2.4, 95% CI: 1.2–4.7; p = 0.013) (114).

A meta-analysis of six studies involving 1,123 patients with NSCLC, mRCC, and melanoma showed that probiotic supplementation with various strains was associated with a significant improvement in OS (HR: 0.527) and ORR (OR: 2.831), with trends toward improved PFS and DCR (85). Subgroup analysis in NSCLC patients receiving ICIs and probiotics demonstrated significantly longer PFS (HR: 0.532; 95% CI: 0.354–0.798; p = 0.002), improved OS (HR: 0.528; 95% CI: 0.306–0.912; p = 0.022), higher ORR (OR: 2.552; 95% CI: 1.279–5.091; p = 0.008), and DCR (OR: 2.439; 95% CI: 1.534–3.878; p < 0.001). An updated meta-analysis including 8 retrospective and 4 prospective studies with 3,142 patients further confirmed that probiotics significantly prolonged OS (HR: 0.58 0.54–0.81; p < 0.001) and PFS (HR: 0.66 0.54–0.81; p < 0.001), and improved ORR (OR: 1.75; 1.27–2.40; p = 0.001) and DCR (OR: 1.93; 1.11–3.35; p = 0.002). Notably, in NSCLC patients exposed to antibiotics, probiotics mitigated the negative effects on OS (HR: 0.45; 0.34–0.59; p < 0.001) and PFS (HR: 0.48; 95% CI: 0.38–0.62; p < 0.001) (16).

While these findings suggest a potential benefit of probiotic supplementation in enhancing ICI efficacy, most studies to date are retrospective, with small sample sizes and heterogeneous study designs (**Table 2**). Additionally, it is important to note that majority of clinical studies evaluated a wide range of commercially available probiotics, often multi-strain formulas or single-strain agents, without standardization. For example, in the studies by Morita et al. and Takada et al., patients received a heterogeneous mix of probiotic products such as Clostridium butyricum, Bifidobacterium spp., and antibiotic-resistant lactic acid bacteria (108, 109). Similarly, in the prospective lung cancer study by Tong et al., patients used diverse multi-strain formulations including *Lactobacillus acidophilus, Bifidobacterium lactis, Streptococcus thermophilus, Bacillus subtilis, Enterococcus faecium, Lactobacillus rhamnosus*, and *Bacillus licheniformi* according to product instructions, starting with immunotherapy and continuing until progression (110). In contrast, some studies, such as that by Ebrahimi et al., used a defined dosing regimen with CBM588 at 80 mg twice daily (113).

**Table 2:** Clinical studies supporting Probiotics- ICI synergy.

Title		Location	Type of study	Cancer of focus	Probiotic	Results	
Association of Probiotic Clostridium butyricum Therapy with Survival and Response to Immune Checkpoint Blockade in Patients with Lung Cancer.		Japan	Retrospective study	Non-Small Cell Lung Cancer (NSCLC)	Clostridium butyricum (MIYAIRI 588 strain)	mPFS 250 vs. 101 days; HR 0.37, P = 0.001 OS not reached vs. 361 days; HR 0.20, P < 0.001.	
Impacts of probiotics on the efficacies of immune checkpoint inhibitors with or without chemotherapy for patients with advanced non-small-cell lung cancer.		Japan	Retrospective study	NSCLC	Oral probiotics (strain not specified)	ICI monotherapy: mPFS 7.9 vs. 2.9 months; HR 0.54, p <.001. OS not reached vs. 13.1 months; HR 0.45, p <.001. ICI + chemotherapy: mPFS 8.8 vs. 8.6 months; HR 0.89, p = 0.43. OS not reached vs. 22.6 months; HR 0.61, p = 0.03.	
Clinical impact of probiotics on the efficacy of anti-PD-1 monotherapy in patients with nonsmall cell lung cancer: A multicenter retrospective survival analysis study with inverse probability of treatment weighting.		Japan	Retrospective study	NSCLC	Oral probiotics (strain not specified)	Overall Response Rate: OR 0.43, 95% CI 0.29- 0.63, p <.0001.	
Concomitant Medications Alter Clinical Outcomes in Patients with Advanced Digestive Tract Cancer Receiving PD-1 Checkpoint Inhibitors Combined with Antiangiogenetic Agents.		China	Retrospective study	Hepatocellular carcinoma, Colorectal cancer, Gastric cancer	Oral probiotics (strain not specified)	Overall Response Rate: OR 2.4, 95% CI 1.2–4.7, p = 0.013.	
Evaluating Oral Probiotic Supplements as Complementary Treatment in Advanced Lung Cancer Patients Receiving ICIs: A Prospective Real-World Study.		China	Prospective study	Advanced Lung Cancer (SCLC and NSCLC)	Oral probiotics (strain not specified)	SCLC: mPFS: 11.1 months vs 7.0 months, p = 0.049. NSCLC: mPFS: 16.5 months vs 12.3 months, p = 0.56. Overall Response Rate 57.7% vs 58.1%, p>0.26.	
Correlation of distribution characteristics and dynamic changes of gut microbiota with the efficacy of immunotherapy in EGFR-mutated non-small cell lung cancer.		China	Prospective study	EGFR-mutant NSCLC	NA	Antibiotic use: mPFS 4.8 months vs 6.7 months; hazard ratio 3.18; 95% CI 1.48- 6.85; p= 0.003. OS: 7.4 *vs*. 16.1 months; HR 3.64; 95% CI 1.30- 10.17; p = 0.014.	
Gut microbiome modulates response to anti-PD-1 immunotherapy in melanoma patients.		USA	Prospective study	Melanoma	Ruminococcaceae	Anti-PD-1 responders had significantly higher gut microbiome alpha diversity (P < 0.01) and greater abundance of Ruminococcaceae (P < 0.01).	
Predicting Response to Immunotherapy for Melanoma With Gut Microbiome and Metabolomics (PRIMM).		UK, Netherlands	Prospective study	Melanoma	A panel of species, including Bifidobacterium pseudocatenulatum, Roseburia spp. and Akkermansia muciniphila	Baseline gut microbiome composition predicted immunotherapy response in melanoma in the UK cohort (P = 0.05) but not in the Netherlands cohort, with no significant association for 12-month PFS in either group.	
A Multicenter Phase 1b Randomized, Placebo-controlled, Blinded Study to Evaluate the Safety, Tolerability and Efficacy of Microbiome Study Intervention Administration in Combination With Anti-PD-1 Therapy in Adult Patients With Unresectable or Metastatic Melanoma.	USA		Phase 1b Clinical Trial	Unresectable or Metastatic Melanoma	SER-401: Ruminococcaceae and other spore-forming microbes	Analysis of 14 accrued patients demonstrated that SER-401 + nivolumab arm showed a 25% overall response rate, while the placebo + nivolumab arm showed 67%, but the study was underpowered due to poor accrual.
Cabozantinib and nivolumab with or without live bacterial supplementation in metastatic renal cell carcinoma: a randomized phase 1 trial.	USA		Phase 1 Clinical Trial	Metastatic renal cell carcinoma	CBM588 (bifidogenic probiotic)	Objective response rate 74% compared to 20%, mPFS of 84% versus 60% at 6 months. No significant changes in the abundance of Bifidobacterium spp. or overall gut bacterial diversity after 13 weeks of treatment. However, there was a specific increase in Ruminococcaceae in the experimental arm
Nivolumab plus ipilimumab with or without live bacterial supplementation in metastatic renal cell carcinoma: a randomized phase 1 trial.	USA		Phase 1 clinical trial	Metastatic renal cell carcinoma	CBM588 (bifidogenic probiotic)	PFS 12.7 months compared to 2.5 months with a HR of 0.15 (p < 0.001). Overall survival was not reached. ORR 58% compared to 20%, though this difference was not statistically significant (P = 0.06).
Neoadjuvant nivolumab or nivolumab plus ipilimumab in operable non-small cell lung cancer: the phase 2 randomized NEOSTAR trial.	USA		Phase 2 clinical trial	NSCLC	Ruminococcus and Akkermansia spp.	Akkermansia sp. (correlation coefficient R = 0.44, p = 0.05) and Bifidobacterium sp. (R = 0.47, p = 0.04) were positively correlated with Major Pathologic Response (MPR).
The correlation between probiotic use and outcomes of cancer patients treated with immune checkpoint inhibitors.	China		Meta-analysis	Cancer (overall with subgroup analysis)	Oral probiotics (strain not specified)	PFS: HR: 0.585, 95% CI: 0.328–1.045, p = 0.070. OS: HR: 0.526, 95% CI: 0.341–0.812, p = 0.004. ORR: OR: 2.831, 95% CI: 1.578–5.076, p < 0.001.
Assessing the impact of probiotics on immunotherapy effectiveness and antibiotic-mediated resistance in cancer: a systematic review and meta-analysis	UK and China		Meta-analysis	Cancer (overall with subgroup analysis)	Oral probiotics (strain not specified)	PFS: HR = 0.66, 95% CI: 0.54–0.81, p < 0.001. OS: HR = 0.58, 95% CI: 0.46–0.73, p < 0.001. ORR: OR = 1.75, 95% CI: 1.27–2.40, p = 0.001.

### 3.4 Probiotics and immune-related adverse events

IrAEs are common toxicities associated with ICIs and are thought to reflect immune system overactivation. Probiotics have emerged as a potential strategy to modulate these responses (**Table 3**) (115, 116). Certain microbial signatures have been associated with either increased or reduced irAE risk. Firmicutes have been linked to higher irAE rates, whereas *Bacteroidetes* spp.*, Bifidobacterium* spp.*, and Desulfovibrio* spp. may have protective effects (117). In murine models, *Bifidobacterium* spp. supplementation reduced ICI-induced colitis by increasing Tregs and enhancing Lactobacillus abundance, while Lactobacillus resolved colitis by reducing activation of innate lymphoid cells (116). Findings such as these highlight the importance of rigorous clinical and translational studies to better understand the optimal application of probiotics.

**Table 3:** Studies on Probiotics and Immune-Related Adverse Events (IrAEs).

Title	Location	Type of study	IrAE	Probiotic	Results
Faecalibacterium prausnitzii Abrogates Intestinal Toxicity and Promotes Tumor Immunity to Increase the Efficacy of Dual CTLA4 and PD-1 Checkpoint Blockade.	China	Mouse preclinical study	ICI-induced colitis	*Faecalibacterium prausnitzii*	F. prausnitzii mitigated ICI-induced colitis in preclinical mouse models by reducing inflammation and immune cell infiltration. Patients receiving ICIs who developed colitis had lower abundance of F. prausnitzii in their gut microbiota.
Correlation of the Gut Microbiome and Immune-Related Adverse Events in Gastrointestinal Cancer Patients Treated with Immune Checkpoint Inhibitors.	China	Prospective study	IrAEs in gastrointestinal cancer	Ruminococcus callidus and Bacteroides xylanisolvens	Ruminococcus callidus and Bacteroides xylanisolvens were enriched in patients without severe irAEs, while Bifidobacterium dentium, Rothia mucilaginosa, and Gemella haemolysans were significantly higher in irAE patients.
The Gut Microbiome Associates with Immune Checkpoint Inhibition Outcomes in Patients with Advanced Non-Small Cell Lung Cancer.	Japan	Prospective study	IrAEs in Non-Small Cell Lung Cancer	*Ruminococcaceae* UCG 13 and *Agathobacter*	Ruminococcaceae UCG 13 was enriched in patients with OS > 12 months. Agathobacter was enriched in patients with favorable ORR and PFS > 6 months.
Prospective correlation between the patient microbiome with response to and development of immune-mediated adverse effects to immunotherapy in lung cancer.	USA	Prospective study	IrAEs in Lung Cancer	Bifidobacterium spp. and Desulfovibrio spp.	Of 34 lung cancer patients, Bifidobacterium spp. (p = 0.001) and Desulfovibrio spp. (p = 0.0002) were enriched in patients without immune-related adverse events (irAEs), while Clostridiales (p = 0.018) and Rikenellaceae (p = 0.016) were increased in responders to chemoimmunotherapy.

Clinical data on probiotics and irAEs remain mixed. In the large retrospective study by Morita et al., probiotic use was not associated with a higher incidence of irAEs in patients on ICI monotherapy (108). However, among those receiving combination therapy with chemotherapy, increased rates of colitis and hypophysitis were reported. No significant associations were observed for other irAEs such as hypothyroidism, pneumonitis, hepatitis, or nephritis, though small subgroup sizes may have limited the findings. Other microbes, such as Faecalibacterium prausnitzii, have also been studied for their anti-inflammatory properties. In a murine model, F. prausnitzii alleviated ICI-induced colitis while preserving or even enhancing antitumor immunity, marked by increased tumor-infiltrating T cells and minimal systemic inflammation. Notably, patients who developed ICI-induced colitis had lower baseline levels of F. prausnitzii (118). A prospective study of 95 patients with gastrointestinal cancers treated with ICIs found that higher levels of *Lactobacillus* spp. *and Bifidobacterium* spp. correlated with fewer irAEs in gastric cancer (119). Similar findings have been reported in NSCLC, where higher abundance of these genera was associated with reduced incidence or severity of irAEs (117, 120, 121).

## 4 Integrative approaches to study probiotics and immunotherapy

Advances in microbiome sequencing, including 16S rRNA and metagenomic shotgun approaches, have consistently revealed distinct microbial signatures between responders and non-responders of ICI, supporting microbiome profiling as a promising tool for patient stratification and predictive biomarker development in immunotherapy (122, 123). However, to move beyond correlative associations and define causality, it is critical to establish a robust framework of mechanistic assays and models. Elucidating how probiotics influence immune responses to checkpoint blockade requires dynamic and longitudinal investigation. Single-time-point assessments fall short in capturing the evolving interplay between the gut microbiota and the host immune system during ICI therapy. Notably, longitudinal studies have shown that key microbial and immune differences between responders and non-responders may only emerge after treatment initiation (124). Thus, serial monitoring of the microbiome and immune parameters is essential to understand the timing, durability, and infer causality of probiotic-induced effects.

Integrating these longitudinal data with experimental platforms such as ex vivo co-culture systems, germ-free animal models, organ-on-chip technologies, and multi-omics profiling can provide high-resolution insight into host–microbe–immune interactions. These complementary tools allow researchers to dissect functional mechanisms, evaluate safety, and identify optimal intervention points. Ultimately, this systems-level approach offers a roadmap for developing microbiome-based adjuvants or biomarkers that enhance ICI efficacy and aligns with the broader goals of precision oncology.

### 4.1 *Ex vivo* immune assays

Mechanistic insights into probiotic and ICI interactions can be initially explored through ex vivo immune assays that replicate tumor–microbe–immune cell interactions in controlled settings. These assays involve co-culturing patient-derived immune cells, such as peripheral blood mononuclear cells or T lymphocytes, with tumor cells, followed by the introduction of selected probiotic strains or their metabolites (125, 126). This approach allows direct evaluation of how probiotics influence immune activation, including cytokine secretion and tumor cell cytotoxicity, outside the complexity of a living organism. Outputs such as T cell proliferation, cytotoxic activity, and cytokine levels (e.g., IFN-γ, IL-2, TNF-α) can be measured to identify probiotic strains with the strongest anti-tumor effects (126, 127). These ex vivo assays offer a rapid and informative readout of probiotic-driven immune modulation and help guide the design of follow-up *in vivo* studies.

### 4.2 Animal models with defined microbiota

Findings from cell-based assays can be expanded using animal models, particularly germ-free or gnotobiotic mice. Germ-free mice offer a sterile gut environment that allows for controlled colonization with specific probiotic strains or defined microbial communities. Researchers can then evaluate tumor responses to ICIs within this tailored microbial landscape (128, 129). Seminal studies have demonstrated that commensal bacteria can significantly influence the efficacy of immunotherapy. Sivan et al. (2015) found that mice colonized with *Bifidobacterium* spp. exhibited enhanced tumor control during anti–PD-L1 treatment (71). Similarly, Vétizou et al. (2015) showed that Bacteroides species were essential for optimal responses to anti–CTLA-4 therapy (101). More recent work has confirmed that fecal microbiota transplants from ICI-responsive cancer patients into germ-free or antibiotic-treated mice produce stronger anti-tumor effects than transplants from non-responders, emphasizing the microbiome’s role in modulating immune responses. Expanding on these findings, germ-free mice can be colonized with candidate probiotic strains to assess their influence on tumor growth, ICI efficacy, and immune cell infiltration *in vivo* (72, 130, 131). These models allow for mechanistic end-point analyses, such as measuring changes in intratumoral T cell activation or myeloid cell phenotypes in the tumor microenvironment (132–134). Overall, the use of germ-free mice colonized with defined microbiota offers a powerful and tightly controlled *in vivo* system to study the therapeutic and immunological impacts of probiotics in cancer immunotherapy.

### 4.3 Gut-on-a-chip microfluidic models

Animal models can be complemented by advanced microphysiological systems that help bridge the gap between mouse and human biology. Gut-on-a-chip technology offers a high-fidelity *in vitro* platform that simulates the human intestinal ecosystem, enabling controlled investigation of microbiota and immune system interactions. These microfluidic devices culture living human intestinal epithelial cells under dynamic flow conditions and can support co-culture with live bacteria for extended periods (135). Gut-on-a-chip models replicate key aspects of the intestinal environment, including three-dimensional tissue architecture, mechanical forces similar to peristalsis, and oxygen gradients. This setup allows for long-term maintenance of both host cells and commensal microbes in close proximity (135). Incorporation of immune components, such as circulating or tissue-resident immune cells, enables the recreation of the intestinal mucosal immune interface. For example, placing peripheral immune cells in a basal channel to represent the lamina propria has demonstrated that exposure to bacterial molecules like lipopolysaccharide triggers cytokine responses (IL-8, IL-6, IL-1β, TNF-α) that closely mimic *in vivo* inflammation (135). This platform allows researchers to introduce specific probiotic strains or their metabolites to human gut epithelium under flow and directly observe resulting changes in immune cell activation, barrier function, and inflammatory signaling. Overall, gut-on-a-chip technology provides a physiologically relevant, human-based model to study probiotic and immune interactions with strong translational potential for clinical applications.

### 4.4 Next-generation sequencing

NGS has become an indispensable tool for uncovering molecular determinants that influence responses to ICIs.Beyond tumor genomics, NGS has also enabled detailed characterization of the gut microbiome to stratify patients based on ICI response. Shotgun metagenomic sequencing of fecal samples has revealed microbial composition and functional potential associated with therapeutic outcomes (136, 137). Notably, genome-resolved metagenomic approaches have uncovered baseline subspecies-level microbial signatures predictive of response, moving beyond strain-level analyses (137). Structural variants (SVs) within bacterial genomes offer an even more granular layer of microbiome heterogeneity, with recent analyses identifying SVs associated with ICI responsiveness, survival outcomes, and irAEs across seven clinical trials involving 996 patients (138). Building on these findings, integrative meta-analyses leveraging NGS data have led to the development of Gut OncoMicrobiome Signatures (GOMS), a standardized framework for identifying reproducible microbial biomarkers across diverse patient cohorts and therapeutic regimens (28). Together, these NGS-enabled approaches provide a comprehensive view of host and microbial factors that shape ICI efficacy, supporting their continued integration into precision oncology strategies.

### 4.5 Multi-omics to map microbiome–immune interactions

Because gut microbiota and host immune interactions span multiple biological layers, a multi-omics approach is essential to fully understand the mechanisms underlying probiotic and ICI interplay. Integrating metagenomics (microbial composition and genes), metatranscriptomics (gene expression), metabolomics (small-molecule metabolites), and proteomics (protein signaling) enables comprehensive mapping of how probiotics influence the host–microbe ecosystem (139, 140). Recent multi-omic studies in immunotherapy patients have identified specific microbial taxa and metabolites linked to treatment outcomes. SCFAs and tryptophan derivatives, such as indoles, have emerged as candidate biomarkers associated with improved ICI responses (139). Combining microbiome sequencing with host immune and metabolic profiling can help pinpoint molecular mediators of probiotic effects. For instance, analyzing stool metagenomes alongside cytokine levels or immune transcriptomes from blood or tumor tissue has revealed associations between specific microbes and metabolic pathways that enhance T cell infiltration into tumors (141).

## 5 Clinical implications and challenges

As the gut microbiome is an emerging critical modulator of immune responses in cancer therapy, integrating microbiome-targeted strategies such as probiotics into immuno-oncology regimens presents a promising yet complex challenge. While preclinical and clinical data support the potential of gut microbiota to enhance ICI efficacy, translating these findings into clinical practice requires careful consideration of numerous confounding variables and implementation barriers.

The gut microbiota is influenced by several host-specific factors, including ethnicity, race, diet, and lifestyle habits (142, 143). Although it remains unclear whether these same variables directly impact the immunomodulatory effects of probiotics, studies suggest that dietary interventions particularly Mediterranean or high-fiber diets can enrich gut microbial diversity and improve immunotherapy outcomes (144). High-fiber diets have been associated with increased populations of SCFA producing bacteria and improved responses to ICIs. For example, melanoma patients consuming high dietary fiber had significantly longer progression-free survival compared to those with low fiber diet or probiotics (84). However, accounting for such lifestyle, geographical and ethnic factors as confounders in clinical trials remains a challenge and highlights the importance of nutritional context in microbiome-targeted interventions.

Beyond host-related factors, inconsistencies across studies may also stem from the lack of standardization in probiotic strain selection, dosage, and administration schedules. Although meta-analyses have not demonstrated major differences among probiotic subgroups, direct head-to-head comparisons of individual strains and consortia remain limited (16, 108). The strain type, relative abundance, and formulation strategy appear to be as critical as the bacterial species itself in determining clinical outcomes. Furthermore, many studies rely on commercially available probiotics, where labeling inaccuracies and suboptimal viability of the declared strains raise additional concerns and introduce further variability (145). The absence of unified regulatory frameworks governing probiotic formulation, strain identity, and potency contributes to wide heterogeneity across products and supplements. Therefore, probiotic use in cancer immunotherapy should be approached with caution outside rigorously controlled research settings. Only a few prospective studies, such as those evaluating CBM588 in renal cell carcinoma, have applied consistent probiotic regimens (112, 113). Rational design of defined microbial consortia with validated immunostimulatory properties is a major area of ongoing research.

Probiotics are generally well tolerated, yet safety data in oncology populations remain sparse. In a clinical study assessing CBM588 alongside ICIs, common grade 3 or higher toxicities included transaminitis (10%), hypertension (7%), and diarrhea (7%), with no significant difference between arms (113). However, emerging concerns suggest that indiscriminate use of over-the-counter probiotics may reduce microbial diversity or interfere with endogenous commensals (84, 146–150). Certain strains may fail to durably engraft or even compete with native microbes, potentially undermining the very immune mechanisms that ICIs aim to stimulate. One prevailing hypothesis is that single- or limited-strain supplements may outcompete beneficial resident taxa, reducing microbial functional capacity and compromising antitumor immunity (151).

Another critical variable is the effect of concurrent medications. Antibiotic use is known to impair ICI efficacy, but other commonly prescribed agents such as corticosteroids, proton pump inhibitors (PPIs), NSAIDs, statins, and metformin may also modulate immune responses (111, 152). Notably, PPIs have been linked to shorter OS in patients receiving ICIs for melanoma and NSCLC (152, 153). However, a contrasting study suggested that therapeutic, but not prophylactic, PPI use might improve outcomes (114). These findings emphasize the need to carefully account for medication history in any clinical trial evaluating the efficacy of probiotics alongside immunotherapy.

While immunomodulation by probiotics likely involves a network of interactions spanning multiple signaling pathways, immune cell subsets, and microbial metabolites, mechanistic understanding of probiotics’ effects in cancer immunotherapy remains incomplete. These complexities make it difficult to attribute therapeutic effects to any single microbe or metabolite. Additionally, many existing studies capture only static microbiome snapshots, missing the dynamic shifts that occur throughout the treatment course. Longitudinal sampling combined with multi-omics integration, and functional immune assays will be essential to clarify the evolving host–microbe–immune landscape over time (139, 140).

Looking forward, future trials must be thoughtfully designed to address these challenges. Trials should incorporate longitudinal multi-omics sampling to assess dynamic shifts in microbiota and immune parameters, use standardized and well-characterized probiotic formulations, and account for confounding variables such as diet, lifestyle, and concurrent medications. Stratification based on baseline microbiome profiles or responder-associated microbial signatures may enhance patient selection. Multi-center collaboration and harmonized protocols will help validate findings across populations and cancer types. However, logistical barriers remain: probiotics pose limited patentability, which dampens industry incentives, and investigational new drug (IND) applications require complex chemistry, manufacturing, and controls (CMC) documentation that is rarely available for probiotics sold as food supplements. These regulatory and economic constraints have contributed to a lack of pharmaceutical investment in this space. Consequently, academic institutions, non-profit organizations, and public–private partnerships may need to lead efforts in clinical development to ensure rigorous testing and broader access. By embracing a systematic approach that integrates clinical, microbial, and immunological data, future studies can provide actionable insights into how best to leverage the microbiome to improve cancer immunotherapy outcomes.

## 6 Conclusion

The gut microbiome is now widely recognized as a critical modulator of immune responses to cancer therapy, particularly ICIs. Among microbiome-targeted strategies, probiotics have emerged as a promising potential adjunct to enhance both the efficacy and safety of ICIs. Preclinical and early-phase clinical studies have shown that specific strains - such as *Bifidobacterium* spp.*, Lactobacillus* spp.*, Clostridium butyricum*, and *Akkermansia muciniphila* can augment dendritic cell activation, boost cytotoxic T-cell function, strengthen gut barrier integrity, and counteract dysbiosis-associated resistance. Retrospective and prospective studies across multiple tumor types similarly suggest improved response rates and survival in patients receiving probiotics alongside ICIs.

However, unmeasured confounders, including diet, antibiotic exposure, ethnicity, and baseline microbiome composition, may influence these associations in retrospective and meta-analytic data. Moreover, the precise mechanisms driving probiotic-mediated benefits in prospective settings remain to be fully elucidated. Based on current evidence, probiotic supplementation should be considered only within the context of rigorously designed clinical trials for patients undergoing immunotherapy. As enthusiasm for probiotic–ICI synergy continues to grow, translating these findings into practice will require coordinated, high-quality efforts integrating longitudinal microbiome profiling, immune monitoring, and randomized clinical trials to define optimal strains, dosing, and patient selection. With a more complete understanding of host–microbiome–immune interactions, probiotics hold the potential to become scalable, cost-effective, and personalized tools to optimize immunotherapy outcomes.

## References

[1] Shiravand Y Khodadadi F Kashani SMA Hosseini-Fard SR Hosseini S Sadeghirad H . Immune checkpoint inhibitors in cancer therapy. Curr Oncol. (2022) 29:3044–60. doi: 10.3390/curroncol29050247, PMID: 35621637 PMC9139602

[2] Yan Y Kumar AB Finnes H Markovic SN Park S Dronca RS . Combining immune checkpoint inhibitors with conventional cancer therapy. Front Immunol. (2018) 9:1739. doi: 10.3389/fimmu.2018.01739, PMID: 30100909 PMC6072836

[3] Buchbinder EI Desai A . CTLA-4 and PD-1 pathways: Similarities, differences, and implications of their inhibition. Am J Clin Oncol. (2016) 39:98–106. doi: 10.1097/COC.0000000000000239, PMID: 26558876 PMC4892769

[4] Tan CL Kuchroo JR Sage PT Liang D Francisco LM Buck J . PD-1 restraint of regulatory T cell suppressive activity is critical for immune tolerance. J Exp Med. (2021) 218:e20182232. doi: 10.1084/jem.20182232, PMID: 33045061 PMC7543091

[5] Larkin J Chiarion-Sileni V Gonzalez R Grob JJ Cowey CL Lao CD . Combined nivolumab and ipilimumab or monotherapy in untreated melanoma. N Engl J Med. (2015) 373:23–34. doi: 10.1056/NEJMoa1504030, PMID: 26027431 PMC5698905

[6] Menon S Shin S Dy G . Advances in cancer immunotherapy in solid tumors. Cancers (Basel). (2016) 8:106. doi: 10.3390/cancers8120106, PMID: 27886124 PMC5187504

[7] Farkona S Diamandis EP Blasutig IM . Cancer immunotherapy: the beginning of the end of cancer? BMC Med. (2016) 14:73. doi: 10.1186/s12916-016-0623-5, PMID: 27151159 PMC4858828

[8] Zhang C Zhang C Wang H . Immune-checkpoint inhibitor resistance in cancer treatment: Current progress and future directions. Cancer Lett. (2023) 562:216182. doi: 10.1016/j.canlet.2023.216182, PMID: 37076040

[9] Bai R Chen N Li L Du N Bai L Lv Z . Mechanisms of cancer resistance to immunotherapy. Front Oncol. (2020) 10:1290. doi: 10.3389/fonc.2020.01290, PMID: 32850400 PMC7425302

[10] Zhou B Gao Y Zhang P Chu Q . Acquired resistance to immune checkpoint blockades: The underlying mechanisms and potential strategies. Front Immunol. (2021) 12:693609. doi: 10.3389/fimmu.2021.693609, PMID: 34194441 PMC8236848

[11] Alsaafeen BH Ali BR Elkord E . Resistance mechanisms to immune checkpoint inhibitors: updated insights. Mol Cancer. (2025) 24:20. doi: 10.1186/s12943-024-02212-7, PMID: 39815294 PMC11734352

[12] Berland L Gabr Z Chang M Ilié M Hofman V Rignol G . Further knowledge and developments in resistance mechanisms to immune checkpoint inhibitors. Front Immunol. (2024) 15:1384121. doi: 10.3389/fimmu.2024.1384121, PMID: 38903504 PMC11188684

[13] Reynoso-García J Miranda-Santiago AE Meléndez-Vázquez NM Acosta-Pagán K Sánchez-Rosado M Díaz-Rivera J . A complete guide to human microbiomes: Body niches, transmission, development, dysbiosis, and restoration. Front Syst Biol. (2022) 2:951403. doi: 10.3389/fsysb.2022.951403, PMID: 38993286 PMC11238057

[14] Whiteside SA McGinniss JE Collman RG . The lung microbiome: progress and promise. J Clin Invest. (2021) 131:e150473. doi: 10.1172/JCI150473, PMID: 34338230 PMC8321564

[15] Jost M Wehkamp U . The skin microbiome and influencing elements in cutaneous T-cell lymphomas. Cancers (Basel). (2022) 14:1324. doi: 10.3390/cancers14051324, PMID: 35267632 PMC8909499

[16] Zhao S Lu Z Zhao F Tang S Zhang L Feng C . Assessing the impact of probiotics on immunotherapy effectiveness and antibiotic-mediated resistance in cancer: a systematic review and meta-analysis. Front Immunol. (2025) 16:1538969. doi: 10.3389/fimmu.2025.1538969, PMID: 40191197 PMC11968366

[17] Thursby E Juge N . Introduction to the human gut microbiota. Biochem J. (2017) 474:1823–36. doi: 10.1042/BCJ20160510, PMID: 28512250 PMC5433529

[18] Jandhyala SM Talukdar R Subramanyam C Vuyyuru H Sasikala M Nageshwar Reddy D . Role of the normal gut microbiota. World J Gastroenterol. (2015) 21:8787–803. doi: 10.3748/wjg.v21.i29.8787, PMID: 26269668 PMC4528021

[19] Li Z Xiong W Liang Z Wang J Zeng Z Kołat D . Critical role of the gut microbiota in immune responses and cancer immunotherapy. J Hematol Oncol. (2024) 17:33. doi: 10.1186/s13045-024-01541-w, PMID: 38745196 PMC11094969

[20] Baruch EN Youngster I Ben-Betzalel G Ortenberg R Lahat A Katz L . Fecal microbiota transplant promotes response in immunotherapy-refractory melanoma patients. Science. (2021) 371:602–9. doi: 10.1126/science.abb5920, PMID: 33303685

[21] Petersen C Round JL . Defining dysbiosis and its influence on host immunity and disease: How changes in microbiota structure influence health. Cell Microbiol. (2014) 16:1024–33. doi: 10.1111/cmi.12308, PMID: 24798552 PMC4143175

[22] PrasadR RehmanA RehmanL DarbaniyanF BlumenbergV SchubertM-L . Antibiotic-induced loss of gut microbiome metabolic output correlates with clinical responses to CAR T-cell therapy. Blood. (2025) 145:823–39. doi: 10.1182/blood.2024025366, PMID: 39441941

[23] Aghamajidi A Maleki Vareki S . The effect of the gut Microbiota on systemic and anti-tumor immunity and response to systemic therapy against cancer. Cancers (Basel). (2022) 14:3563. doi: 10.3390/cancers14153563, PMID: 35892821 PMC9330582

[24] Zhang H Pan Y Jiang Y Chen M Ma X Yu X . Akkermansia muciniphila ONE effectively ameliorates dextran sulfate sodium (DSS)-induced ulcerative colitis in mice. NPJ Sci Food. (2024) 8:97. doi: 10.1038/s41538-024-00339-x, PMID: 39562574 PMC11576909

[25] Naqash AR Kihn-Alarcón AJ Stavraka C Kerrigan K Maleki Vareki S Pinato DJ . The role of gut microbiome in modulating response to immune checkpoint inhibitor therapy in cancer. Ann Transl Med. (2021) 9:1034. doi: 10.21037/atm-20-6427, PMID: 34277834 PMC8267312

[26] Bouferraa Y Chedid A Amhaz G El Lakkiss A Mukherji D Temraz S . The role of gut Microbiota in overcoming resistance to checkpoint inhibitors in cancer patients: Mechanisms and challenges. Int J Mol Sci. (2021) 22:8036. doi: 10.3390/ijms22158036, PMID: 34360802 PMC8347208

[27] Blake SJ Wolf Y Boursi B Lynn DJ . Role of the microbiota in response to and recovery from cancer therapy. Nat Rev Immunol. (2024) 24:308–25. doi: 10.1038/s41577-023-00951-0, PMID: 37932511

[28] Thomas AM Fidelle M Routy B Kroemer G Wargo JA Segata N . Gut OncoMicrobiome Signatures (GOMS) as next-generation biomarkers for cancer immunotherapy. Nat Rev Clin Oncol. (2023) 20:583–603. doi: 10.1038/s41571-023-00785-8, PMID: 37365438 PMC11258874

[29] Panigrahi P Parida S Nanda NC Satpathy R Pradhan L Chandel DS . A randomized synbiotic trial to prevent sepsis among infants in rural India. Nature. (2017) 548:407–12. doi: 10.1038/nature23480, PMID: 28813414

[30] Qiu Y Dizman N Jiang Y Robert M Farias R Levy EJ . Clinical outcomes of the DIET study: A randomized controlled phase 2 trial of a high fiber diet intervention (HFDI) in patients with melanoma receiving immune checkpoint blockade (ICB). J Clin Oncol. (2025) 43:9511–1. doi: 10.1200/JCO.2025.43.16_suppl.9511

[31] Elkrief A Routy B Derosa L Bolte L Wargo JA McQuade JL . Gut Microbiota in immuno-oncology: A practical guide for medical oncologists with a focus on antibiotics stewardship. Am Soc Clin Oncol Educ Book. (2025) 45:e472902. doi: 10.1200/EDBK-25-472902, PMID: 40262063

[32] Sommer F Bäckhed F . The gut microbiota–masters of host development and physiology. Nat Rev Microbiol. (2013) 11:227–38. doi: 10.1038/nrmicro2974, PMID: 23435359

[33] Nicholson JK Holmes E Kinross J Burcelin R Gibson G Jia W . Host-gut microbiota metabolic interactions. Science. (2012) 336:1262–7. doi: 10.1126/science.1223813, PMID: 22674330

[34] Rooks MG Garrett WS . Gut microbiota, metabolites and host immunity. Nat Rev Immunol. (2016) 16:341–52. doi: 10.1038/nri.2016.42, PMID: 27231050 PMC5541232

[35] Liu X Chen Y Zhang S Dong L . Gut microbiota-mediated immunomodulation in tumor. J Exp Clin Cancer Res. (2021) 40:221. doi: 10.1186/s13046-021-01983-x, PMID: 34217349 PMC8254267

[36] Peterson DA Frank DN Pace NR Gordon JI . Metagenomic approaches for defining the pathogenesis of inflammatory bowel diseases. Cell Host Microbe. (2008) 3:417–27. doi: 10.1016/j.chom.2008.05.001, PMID: 18541218 PMC2872787

[37] Vimal J Himal I Kannan S . Role of microbial dysbiosis in carcinogenesis & cancer therapies. Indian J Med Res. (2020) 152:553–61. doi: 10.4103/ijmr.IJMR_1026_18, PMID: 34145094 PMC8224166

[38] Cui B Luo H He B Liu X Lv D Zhang X . Gut dysbiosis conveys psychological stress to activate LRP5/β-catenin pathway promoting cancer stemness. Signal Transduct Target Ther. (2025) 10:79. doi: 10.1038/s41392-025-02159-1, PMID: 40038255 PMC11880501

[39] Maslowski KM Mackay CR . Diet, gut microbiota and immune responses. Nat Immunol. (2011) 12:5–9. doi: 10.1038/ni0111-5, PMID: 21169997

[40] Bemark M Pitcher MJ Dionisi C Spencer J . Gut-associated lymphoid tissue: a microbiota-driven hub of B cell immunity. Trends Immunol. (2024) 45:211–23. doi: 10.1016/j.it.2024.01.006, PMID: 38402045 PMC11227984

[41] Alford SK Longmore GD Stenson WF Kemper C . CD46-induced immunomodulatory CD4+ T cells express the adhesion molecule and chemokine receptor pattern of intestinal T cells. J Immunol. (2008) 181:2544–55. doi: 10.4049/jimmunol.181.4.2544, PMID: 18684945 PMC2597161

[42] Fidelle M Tian A-L Zitvogel L Kroemer G . Bile acids regulate MAdCAM-1 expression to link the gut microbiota to cancer immunosurveillance. Oncoimmunology. (2023) 12:2224672. doi: 10.1080/2162402X.2023.2224672, PMID: 37405191 PMC10316723

[43] Fidelle M Rauber C Alves Costa Silva C Tian A-L Lahmar I de la Varende A-LM . A microbiota-modulated checkpoint directs immunosuppressive intestinal T cells into cancers. Science. (2023) 380:eabo2296. doi: 10.1126/science.abo2296, PMID: 37289890

[44] Helmink BA Khan MAW Hermann A Gopalakrishnan V Wargo JA . The microbiome, cancer, and cancer therapy. Nat Med. (2019) 25:377–88. doi: 10.1038/s41591-019-0377-7, PMID: 30842679

[45] Koh A De Vadder F Kovatcheva-Datchary P Bäckhed F . From dietary fiber to host physiology: Short-chain fatty acids as key bacterial metabolites. Cell. (2016) 165:1332–45. doi: 10.1016/j.cell.2016.05.041, PMID: 27259147

[46] Smith PM Howitt MR Panikov N Michaud M Gallini CA Bohlooly-Y M . The microbial metabolites, short-chain fatty acids, regulate colonic Treg cell homeostasis. Science. (2013) 341:569–73. doi: 10.1126/science.1241165, PMID: 23828891 PMC3807819

[47] Han A Bennett N Ahmed B Whelan J Donohoe DR . Butyrate decreases its own oxidation in colorectal cancer cells through inhibition of histone deacetylases. Oncotarget. (2018) 9:27280–92. doi: 10.18632/oncotarget.25546, PMID: 29930765 PMC6007476

[48] Fellows R Denizot J Stellato C Cuomo A Jain P Stoyanova E . Microbiota derived short chain fatty acids promote histone crotonylation in the colon through histone deacetylases. Nat Commun. (2018) 9:105. doi: 10.1038/s41467-017-02651-5, PMID: 29317660 PMC5760624

[49] Donohoe DR Collins LB Wali A Bigler R Sun W Bultman SJ . The Warburg effect dictates the mechanism of butyrate-mediated histone acetylation and cell proliferation. Mol Cell. (2012) 48:612–26. doi: 10.1016/j.molcel.2012.08.033, PMID: 23063526 PMC3513569

[50] Furusawa Y Obata Y Fukuda S Endo TA Nakato G Takahashi D . Commensal microbe-derived butyrate induces the differentiation of colonic regulatory T cells. Nature. (2013) 504:446–50. doi: 10.1038/nature12721, PMID: 24226770

[51] Luu M Riester Z Baldrich A Reichardt N Yuille S Busetti A . Microbial short-chain fatty acids modulate CD8+ T cell responses and improve adoptive immunotherapy for cancer. Nat Commun. (2021) 12:4077. doi: 10.1038/s41467-021-24331-1, PMID: 34210970 PMC8249424

[52] Roager HM Licht TR . Microbial tryptophan catabolites in health and disease. Nat Commun. (2018) 9:3294. doi: 10.1038/s41467-018-05470-4, PMID: 30120222 PMC6098093

[53] Wang J Zhu N Su X Gao Y Yang R . Gut-Microbiota-derived metabolites maintain gut and systemic immune homeostasis. Cells. (2023) 12:793. doi: 10.3390/cells12050793, PMID: 36899929 PMC10000530

[54] Agus A Planchais J Sokol H . Gut Microbiota regulation of tryptophan metabolism in health and disease. Cell Host Microbe. (2018) 23:716–24. doi: 10.1016/j.chom.2018.05.003, PMID: 29902437

[55] Biagioli M Carino A Cipriani S Francisci D Marchianò S Scarpelli P . The bile acid receptor GPBAR1 regulates the M1/M2 phenotype of intestinal macrophages and activation of GPBAR1 rescues mice from Murine colitis. J Immunol. (2017) 199:718–33. doi: 10.4049/jimmunol.1700183, PMID: 28607110

[56] Fitzgerald GA Terry DS Warren AL Quick M Javitch JA Blanchard SC . Quantifying secondary transport at single-molecule resolution. Nature. (2019) 575:528–34. doi: 10.1038/s41586-019-1747-5, PMID: 31723269 PMC7211416

[57] Rezasoltani S Yadegar A Asadzadeh Aghdaei H Reza Zali M . Modulatory effects of gut microbiome in cancer immunotherapy: A novel paradigm for blockade of immune checkpoint inhibitors. Cancer Med. (2021) 10:1141–54. doi: 10.1002/cam4.3694, PMID: 33369247 PMC7897953

[58] He H Li W Liyanarachchi S Wang Y Yu L Genutis LK . The role of NRG1 in the predisposition to papillary thyroid carcinoma. J Clin Endocrinol Metab. (2018) 103:1369–79. doi: 10.1210/jc.2017-01798, PMID: 29121253 PMC6018707

[59] Gopalakrishnan V Spencer CN Nezi L Reuben A Andrews MC Karpinets TV . Gut microbiome modulates response to anti-PD-1 immunotherapy in melanoma patients. Science. (2018) 359:97–103. doi: 10.1126/science.aan4236, PMID: 29097493 PMC5827966

[60] Matson V Fessler J Bao R Chongsuwat T Zha Y Alegre M-L . The commensal microbiome is associated with anti-PD-1 efficacy in metastatic melanoma patients. Science. (2018) 359:104–8. doi: 10.1126/science.aao3290, PMID: 29302014 PMC6707353

[61] Routy B Le Chatelier E Derosa L Duong CPM Alou MT Daillère R . Gut microbiome influences efficacy of PD-1-based immunotherapy against epithelial tumors. Science. (2018) 359:91–7. doi: 10.1126/science.aan3706, PMID: 29097494

[62] Lee E-S Song E-J Nam Y-D Lee S-Y . Probiotics in human health and disease: from nutribiotics to pharmabiotics. J Microbiol. (2018) 56:773–82. doi: 10.1007/s12275-018-8293-y, PMID: 30353462

[63] Vinderola G Sanders ME Salminen S . The concept of postbiotics. Foods. (2022) 11:1077. doi: 10.3390/foods11081077, PMID: 35454664 PMC9027423

[64] Palaria A Johnson-Kanda I O’Sullivan DJ . Effect of a synbiotic yogurt on levels of fecal bifidobacteria, clostridia, and enterobacteria. Appl Environ Microbiol. (2012) 78:933–40. doi: 10.1128/AEM.05848-11, PMID: 22101054 PMC3272998

[65] Ma L Tu H Chen T . Postbiotics in human health: A narrative review. Nutrients. (2023) 15:291. doi: 10.3390/nu15020291, PMID: 36678162 PMC9863882

[66] Boucher E Plazy C Richard ML Suau A Mangin I Cornet M . Inulin prebiotic reinforces host cancer immunosurveillance via γδ T cell activation. Front Immunol. (2023) 14:1104224. doi: 10.3389/fimmu.2023.1104224, PMID: 36875124 PMC9981629

[67] Asim F Clarke L Donnelly E Jamal FR Piccicacchi LM Qadir M . How do tumours outside the gastrointestinal tract respond to dietary fibre supplementation? BMJ Oncol. (2023) 2:e000107. doi: 10.1136/bmjonc-2023-000107, PMID: 39886510 PMC11203104

[68] Sampsell K Hao D Reimer RA . The gut Microbiota: A potential gateway to improved health outcomes in breast cancer treatment and survivorship. Int J Mol Sci. (2020) 21:9239. doi: 10.3390/ijms21239239, PMID: 33287442 PMC7731103

[69] Han K Nam J Xu J Sun X Huang X Animasahun O . Generation of systemic antitumour immunity via the in *situ* modulation of the gut microbiome by an orally administered inulin gel. Nat BioMed Eng. (2021) 5:1377–88. doi: 10.1038/s41551-021-00749-2, PMID: 34168321 PMC8595497

[70] Rossi M Corradini C Amaretti A Nicolini M Pompei A Zanoni S . Fermentation of fructooligosaccharides and inulin by bifidobacteria: a comparative study of pure and fecal cultures. Appl Environ Microbiol. (2005) 71:6150–8. doi: 10.1128/AEM.71.10.6150-6158.2005, PMID: 16204533 PMC1265942

[71] Sivan A Corrales L Hubert N Williams JB Aquino-Michaels K Earley ZM . Commensal Bifidobacterium promotes antitumor immunity and facilitates anti-PD-L1 efficacy. Science. (2015) 350:1084–9. doi: 10.1126/science.aac4255, PMID: 26541606 PMC4873287

[72] Davar D Dzutsev AK McCulloch JA Rodrigues RR Chauvin J-M Morrison RM . Fecal microbiota transplant overcomes resistance to anti-PD-1 therapy in melanoma patients. Science. (2021) 371:595–602. doi: 10.1126/science.abf3363, PMID: 33542131 PMC8097968

[73] Kim Y Kim G Kim S Cho B Kim S-Y Do E-J . Fecal microbiota transplantation improves anti-PD-1 inhibitor efficacy in unresectable or metastatic solid cancers refractory to anti-PD-1 inhibitor. Cell Host Microbe. (2024) 32:1380–93. doi: 10.1016/j.chom.2024.06.010, PMID: 39059396

[74] Thu MS Ondee T Nopsopon T Farzana IAK Fothergill JL Hirankarn N . Effect of probiotics in breast cancer: A systematic review and meta-analysis. Biol (Basel). (2023) 12:280. doi: 10.3390/biology12020280, PMID: 36829557 PMC10004677

[75] Pala V Sieri S Berrino F Vineis P Sacerdote C Palli D . Yogurt consumption and risk of colorectal cancer in the Italian European prospective investigation into cancer and nutrition cohort. Int J Cancer. (2011) 129:2712–9. doi: 10.1002/ijc.26193, PMID: 21607947

[76] El-Nezami HS Polychronaki NN Ma J Zhu H Ling W Salminen EK . Probiotic supplementation reduces a biomarker for increased risk of liver cancer in young men from Southern China. Am J Clin Nutr. (2006) 83:1199–203. doi: 10.1093/ajcn/83.5.1199, PMID: 16685066

[77] Shida K Nomoto K . Probiotics as efficient immunopotentiators: translational role in cancer prevention. Indian J Med Res. (2013) 138:808–14., PMID: 24434333 PMC3928711

[78] Uccello M Malaguarnera G Basile F D’agata V Malaguarnera M Bertino G . Potential role of probiotics on colorectal cancer prevention. BMC Surg. (2012) 12 Suppl 1:S35. doi: 10.1186/1471-2482-12-S1-S35, PMID: 23173670 PMC3499195

[79] Sivamaruthi BS Kesika P Chaiyasut C . The role of probiotics in colorectal cancer management. Evid Based Complement Alternat Med. (2020) 2020:3535982. doi: 10.1155/2020/3535982, PMID: 32148539 PMC7048916

[80] Yang Y Xia Y Chen H Hong L Feng J Yang J . The effect of perioperative probiotics treatment for colorectal cancer: short-term outcomes of a randomized controlled trial. Oncotarget. (2016) 7:8432–40. doi: 10.18632/oncotarget.7045, PMID: 26824990 PMC4885004

[81] Lee TS . Are probiotics beneficial or harmful for pancreatic cancer outcomes? Probiotics Antimicrob Proteins. (2025) 17:2293–300. doi: 10.1007/s12602-024-10437-7, PMID: 39714574 PMC12405297

[82] Tian Y Li M Song W Jiang R Li YQ . Effects of probiotics on chemotherapy in patients with lung cancer. Oncol Lett. (2019) 17:2836–48. doi: 10.3892/ol.2019.9906, PMID: 30854059 PMC6365978

[83] Yang Z Zhang S Ying L Zhang W Chen X Liang Y . The effect of probiotics supplementation on cancer-treatment complications: a critical umbrella review of interventional meta-analyses. Crit Rev Food Sci Nutr. (2025) 65:3702–27. doi: 10.1080/10408398.2024.2372880, PMID: 39002141

[84] Spencer CN McQuade JL Gopalakrishnan V McCulloch JA Vetizou M Cogdill AP . Dietary fiber and probiotics influence the gut microbiome and melanoma immunotherapy response. Science. (2021) 374:1632–40. doi: 10.1126/science.aaz7015, PMID: 34941392 PMC8970537

[85] Zhang L Jin Q Chai D Kuang T Li C Guan Y . The correlation between probiotic use and outcomes of cancer patients treated with immune checkpoint inhibitors. Front Pharmacol. (2022) 13:937874. doi: 10.3389/fphar.2022.937874, PMID: 36110546 PMC9468893

[86] Derosa L Routy B Desilets A Daillère R Terrisse S Kroemer G . Microbiota-centered interventions: The next breakthrough in immuno-oncology? Cancer Discov. (2021) 11:2396–412. doi: 10.1158/2159-8290, PMID: 34400407

[87] Saito S Okuno A Peng Z Cao D-Y Tsuji NM . Probiotic lactic acid bacteria promote anti-tumor immunity through enhanced major histocompatibility complex class I-restricted antigen presentation machinery in dendritic cells. Front Immunol. (2024) 15:1335975. doi: 10.3389/fimmu.2024.1335975, PMID: 38605963 PMC11008462

[88] Jiang S Ma W Ma C Zhang Z Zhang W Zhang J . An emerging strategy: probiotics enhance the effectiveness of tumor immunotherapy via mediating the gut microbiome. Gut Microbes. (2024) 16:2341717. doi: 10.1080/19490976.2024.2341717, PMID: 38717360 PMC11085971

[89] Tanoue T Morita S Plichta DR Skelly AN Suda W Sugiura Y . A defined commensal consortium elicits CD8 T cells and anti-cancer immunity. Nature. (2019) 565:600–5. doi: 10.1038/s41586-019-0878-z, PMID: 30675064

[90] Li X Zhang S Guo G Han J Yu J . Gut microbiome in modulating immune checkpoint inhibitors. EBioMedicine. (2022) 82:104163. doi: 10.1016/j.ebiom.2022.104163, PMID: 35841869 PMC9297075

[91] Iida N Dzutsev A Stewart CA Smith L Bouladoux N Weingarten RA . Commensal bacteria control cancer response to therapy by modulating the tumor microenvironment. Science. (2013) 342:967–70. doi: 10.1126/science.1240527, PMID: 24264989 PMC6709532

[92] Chen Y Wu J Cai K Xiao X Chen Y Zhang X . Bifidobacterium longum subsp. longum XZ01 delays the progression of colon cancer in mice through the interaction between the microbial spatial distribution and tumour immunity. Int Immunopharmacol. (2025) 150:114283. doi: 10.1016/j.intimp.2025.114283, PMID: 39955918

[93] Shi Y Zheng W Yang K Harris KG Ni K Xue L . Intratumoral accumulation of gut microbiota facilitates CD47-based immunotherapy via STING signaling. J Exp Med. (2020) 217:e20192282. doi: 10.1084/jem.20192282, PMID: 32142585 PMC7201921

[94] Deng H Li Z Tan Y Guo Z Liu Y Wang Y . A novel strain of Bacteroides fragilis enhances phagocytosis and polarises M1 macrophages. Sci Rep. (2016) 6:29401. doi: 10.1038/srep29401, PMID: 27381366 PMC4933912

[95] Chen S Saeed AFUH Liu Q Jiang Q Xu H Xiao GG . Macrophages in immunoregulation and therapeutics. Signal Transduct Target Ther. (2023) 8:207. doi: 10.1038/s41392-023-01452-1, PMID: 37211559 PMC10200802

[96] Lu Y Yuan X Wang M He Z Li H Wang J . Gut microbiota influence immunotherapy responses: mechanisms and therapeutic strategies. J Hematol Oncol. (2022) 15:47. doi: 10.1186/s13045-022-01273-9, PMID: 35488243 PMC9052532

[97] Mager LF Burkhard R Pett N Cooke NCA Brown K Ramay H . Microbiome-derived inosine modulates response to checkpoint inhibitor immunotherapy. Science. (2020) 369:1481–9. doi: 10.1126/science.abc3421, PMID: 32792462

[98] Thapa R Magar AT Shrestha J Panth N Idrees S Sadaf T . Influence of gut and lung dysbiosis on lung cancer progression and their modulation as promising therapeutic targets: a comprehensive review. MedComm. (2024) 5:e70018. doi: 10.1002/mco2.70018, PMID: 39584048 PMC11586092

[99] Hagihara M Kuroki Y Ariyoshi T Higashi S Fukuda K Yamashita R . Clostridium butyricum modulates the microbiome to protect intestinal barrier function in mice with antibiotic-induced dysbiosis. iScience. (2020) 23:100772. doi: 10.1016/j.isci.2019.100772, PMID: 31954979 PMC6970176

[100] Pinato DJ Howlett S Ottaviani D Urus H Patel A Mineo T . Association of prior antibiotic treatment with survival and response to immune checkpoint inhibitor therapy in patients with cancer. JAMA Oncol. (2019) 5:1774–8. doi: 10.1001/jamaoncol.2019.2785, PMID: 31513236 PMC6743060

[101] Vétizou M Pitt JM Daillère R Lepage P Waldschmitt N Flament C . Anticancer immunotherapy by CTLA-4 blockade relies on the gut microbiota. Science. (2015) 350:1079–84. doi: 10.1126/science.aad1329, PMID: 26541610 PMC4721659

[102] Balachandran VP Łuksza M Zhao JN Makarov V Moral JA Remark R . Identification of unique neoantigen qualities in long-term survivors of pancreatic cancer. Nature. (2017) 551:512–6. doi: 10.1038/nature24462, PMID: 29132146 PMC6145146

[103] Duttagupta S Hakozaki T Routy B Messaoudene M . The gut microbiome from a biomarker to a novel therapeutic strategy for immunotherapy response in patients with lung cancer. Curr Oncol. (2023) 30:9406–27. doi: 10.3390/curroncol30110681, PMID: 37999101 PMC10669980

[104] Bender MJ McPherson AC Phelps CM Pandey SP Laughlin CR Shapira JH . Dietary tryptophan metabolite released by intratumoral Lactobacillus reuteri facilitates immune checkpoint inhibitor treatment. Cell. (2023) 186:1846–1862.e26. doi: 10.1016/j.cell.2023.03.011, PMID: 37028428 PMC10148916

[105] Gao G Shen S Zhang T Zhang J Huang S Sun Z . Lacticaseibacillus rhamnosus Probio-M9 enhanced the antitumor response to anti-PD-1 therapy by modulating intestinal metabolites. EBioMedicine. (2023) 91:104533. doi: 10.1016/j.ebiom.2023.104533, PMID: 37027929 PMC10085781

[106] Shi Z Li M Zhang C Li H Zhang Y Zhang L . Butyrate-producing Faecalibacterium prausnitzii suppresses natural killer/T-cell lymphoma by dampening the JAK-STAT pathway. Gut. (2025) 74:557–70. doi: 10.1136/gutjnl-2024-333530, PMID: 39653411 PMC12013593

[107] Tomita Y Ikeda T Sakata S Saruwatari K Sato R Iyama S . Association of probiotic Clostridium butyricum therapy with survival and response to immune checkpoint blockade in patients with lung cancer. Cancer Immunol Res. (2020) 8:1236–42. doi: 10.1158/2326-6066.CIR-20-0051, PMID: 32665261

[108] Morita A Ichihara E Inoue K Fujiwara K Yokoyama T Harada D . Impacts of probiotics on the efficacies of immune checkpoint inhibitors with or without chemotherapy for patients with advanced non-small-cell lung cancer. Int J Cancer. (2024) 154:1607–15. doi: 10.1002/ijc.34842, PMID: 38196128

[109] Takada K Shimokawa M Takamori S Shimamatsu S Hirai F Tagawa T . Clinical impact of probiotics on the efficacy of anti-PD-1 monotherapy in patients with nonsmall cell lung cancer: A multicenter retrospective survival analysis study with inverse probability of treatment weighting. Int J Cancer. (2021) 149:473–82. doi: 10.1002/ijc.33557, PMID: 33720422

[110] Tong L Wan Y Shi X Liu X Liu Z Li Y . Evaluating oral probiotic supplements as complementary treatment in advanced lung cancer patients receiving ICIs: A prospective real-world study. Cancer Control. (2024) 31:10732748241253960. doi: 10.1177/10732748241253959, PMID: 38736182 PMC11089945

[111] Luo W-C Mei S-Q Huang Z-J Chen Z-H Zhang Y-C Yang M-Y . Correlation of distribution characteristics and dynamic changes of gut microbiota with the efficacy of immunotherapy in EGFR-mutated non-small cell lung cancer. J Transl Med. (2024) 22:326. doi: 10.1186/s12967-024-05135-5, PMID: 38566102 PMC10985957

[112] Dizman N Meza L Bergerot P Alcantara M Dorff T Lyou Y . Nivolumab plus ipilimumab with or without live bacterial supplementation in metastatic renal cell carcinoma: a randomized phase 1 trial. Nat Med. (2022) 28:704–12. doi: 10.1038/s41591-022-01694-6, PMID: 35228755 PMC9018425

[113] Ebrahimi H Dizman N Meza L Malhotra J Li X Dorff T . Cabozantinib and nivolumab with or without live bacterial supplementation in metastatic renal cell carcinoma: a randomized phase 1 trial. Nat Med. (2024) 30:2576–85. doi: 10.1038/s41591-024-03086-4, PMID: 38942995 PMC11405272

[114] Wang Y Wu Z Zhu X Zheng Y Yang Y Tu J . Concomitant medications alter clinical outcomes in patients with advanced digestive tract cancer receiving PD-1 checkpoint inhibitors combined with antiangiogenetic agents. J Gastrointest Cancer. (2024) 55:1388–400. doi: 10.1007/s12029-024-01095-7, PMID: 39080229

[115] Das S Johnson DB . Immune-related adverse events and anti-tumor efficacy of immune checkpoint inhibitors. J Immunother Cancer. (2019) 7:306. doi: 10.1186/s40425-019-0805-8, PMID: 31730012 PMC6858629

[116] Tan B Liu Y-X Tang H Chen D Xu Y Chen M-J . Gut microbiota shed new light on the management of immune-related adverse events. Thorac Cancer. (2022) 13:2681–91. doi: 10.1111/1759-7714.14626, PMID: 36043345 PMC9527168

[117] Chau J Yadav M Liu B Furqan M Dai Q Shahi S . Prospective correlation between the patient microbiome with response to and development of immune-mediated adverse effects to immunotherapy in lung cancer. BMC Cancer. (2021) 21:808. doi: 10.1186/s12885-021-08530-z, PMID: 34256732 PMC8278634

[118] Gao Y Xu P Sun D Jiang Y Lin X-L Han T . Faecalibacterium prausnitzii abrogates intestinal toxicity and promotes tumor immunity to increase the efficacy of dual CTLA4 and PD-1 checkpoint blockade. Cancer Res. (2023) 83:3710–25. doi: 10.1158/0008-5472.CAN-23-0605, PMID: 37602831

[119] Zhang Y Cheng S Zou H Han Z Xie T Zhang B . Correlation of the gut microbiome and immune-related adverse events in gastrointestinal cancer patients treated with immune checkpoint inhibitors. Front Cell Infect Microbiol. (2023) 13:1099063. doi: 10.3389/fcimb.2023.1099063, PMID: 37051296 PMC10084768

[120] Hakozaki T Richard C Elkrief A Hosomi Y Benlaïfaoui M Mimpen I . The gut microbiome associates with immune checkpoint inhibition outcomes in patients with advanced non-small cell lung cancer. Cancer Immunol Res. (2020) 8:1243–50. doi: 10.1158/2326-6066.CIR-20-0196, PMID: 32847937

[121] Cascone T William WN Jr Weissferdt A Leung CH Lin HY Pataer A . Neoadjuvant nivolumab or nivolumab plus ipilimumab in operable non-small cell lung cancer: the phase 2 randomized NEOSTAR trial. Nat Med. (2021) 27:504–14. doi: 10.1038/s41591-020-01224-2, PMID: 33603241 PMC8818318

[122] Wensel CR Pluznick JL Salzberg SL Sears CL . Next-generation sequencing: insights to advance clinical investigations of the microbiome. J Clin Invest. (2022) 132:e154944. doi: 10.1172/JCI154944, PMID: 35362479 PMC8970668

[123] Durazzi F Sala C Castellani G Manfreda G Remondini D De Cesare A . Comparison between 16S rRNA and shotgun sequencing data for the taxonomic characterization of the gut microbiota. Sci Rep. (2021) 11:3030. doi: 10.1038/s41598-021-82726-y, PMID: 33542369 PMC7862389

[124] Björk JR Bolte LA Maltez Thomas A Lee KA Rossi N Wind TT . Longitudinal gut microbiome changes in immune checkpoint blockade-treated advanced melanoma. Nat Med. (2024) 30:785–96. doi: 10.1038/s41591-024-02803-3, PMID: 38365950 PMC10957474

[125] Djaldetti M Bessler H . Probiotic strains modulate cytokine production and the immune interplay between human peripheral blood mononucear cells and colon cancer cells. FEMS Microbiol Lett. (2017) 364:3. doi: 10.1093/femsle/fnx014, PMID: 28104778

[126] Bui VT Tseng H-C Kozlowska A Maung PO Kaur K Topchyan P . Augmented IFN-γ and TNF-α induced by probiotic bacteria in NK cells mediate differentiation of stem-like tumors leading to inhibition of tumor growth and reduction in inflammatory cytokine release; Regulation by IL-10. Front Immunol. (2015) 6:576. doi: 10.3389/fimmu.2015.00576, PMID: 26697005 PMC4667036

[127] Gui Q Wang A Zhao X Huang S Tan Z Xiao C . Effects of probiotic supplementation on natural killer cell function in healthy elderly individuals: a meta-analysis of randomized controlled trials. Eur J Clin Nutr. (2020) 74:1630–7. doi: 10.1038/s41430-020-0670-z, PMID: 32514029 PMC7279433

[128] Fiebiger U Bereswill S Heimesaat MM . Dissecting the interplay between intestinal Microbiota and host immunity in health and disease: Lessons learned from germfree and gnotobiotic animal models. Eur J Microbiol Immunol (Bp). (2016) 6:253–71. doi: 10.1556/1886.2016.00036, PMID: 27980855 PMC5146645

[129] Kiousi DE Kouroutzidou AZ Neanidis K Karavanis E Matthaios D Pappa A . The role of the gut microbiome in cancer immunotherapy: Current knowledge and future directions. Cancers (Basel). (2023) 15:2101. doi: 10.3390/cancers15072101, PMID: 37046762 PMC10093606

[130] Crespin A Le Bescop C de Gunzburg J Vitry F Zalcman G Cervesi J . A systematic review and meta-analysis evaluating the impact of antibiotic use on the clinical outcomes of cancer patients treated with immune checkpoint inhibitors. Front Oncol. (2023) 13:1075593. doi: 10.3389/fonc.2023.1075593, PMID: 36937417 PMC10019357

[131] Jain T Sharma P Are AC Vickers SM Dudeja V . New insights into the cancer-microbiome-immune axis: Decrypting a decade of discoveries. Front Immunol. (2021) 12:622064. doi: 10.3389/fimmu.2021.622064, PMID: 33708214 PMC7940198

[132] Yang L Li A Wang Y Zhang Y . Intratumoral microbiota: roles in cancer initiation, development and therapeutic efficacy. Signal Transduct Target Ther. (2023) 8:35. doi: 10.1038/s41392-022-01304-4, PMID: 36646684 PMC9842669

[133] Cao Y Xia H Tan X Shi C Ma Y Meng D . Intratumoural microbiota: a new frontier in cancer development and therapy. Signal Transduct Target Ther. (2024) 9:15. doi: 10.1038/s41392-023-01693-0, PMID: 38195689 PMC10776793

[134] Schmid MC Varner JA . Myeloid cells in the tumor microenvironment: modulation of tumor angiogenesis and tumor inflammation. J Oncol. (2010) 2010:201026. doi: 10.1155/2010/201026, PMID: 20490273 PMC2871549

[135] Valiei A Aminian-Dehkordi J Mofrad MRK . Gut-on-a-chip models for dissecting the gut microbiology and physiology. APL Bioeng. (2023) 7:011502. doi: 10.1063/5.0126541, PMID: 36875738 PMC9977465

[136] Komatsu H Sugimoto T Ogata Y Miura T Aida M Nishiyama H . Characteristics of the gut microbiota in patients with advanced non-small cell lung cancer who responded to immune checkpoint inhibitors. Sci Rep. (2025) 15:23398. doi: 10.1038/s41598-025-08049-4, PMID: 40603595 PMC12222521

[137] Gunjur A Shao Y Rozday T Klein O Mu A Haak BW . A gut microbial signature for combination immune checkpoint blockade across cancer types. Nat Med. (2024) 30:797–809. doi: 10.1038/s41591-024-02823-z, PMID: 38429524 PMC10957475

[138] Liu R Zou Y Wang W-Q Chen J-H Zhang L Feng J . Gut microbial structural variation associates with immune checkpoint inhibitor response. Nat Commun. (2023) 14:7421. doi: 10.1038/s41467-023-42997-7, PMID: 37973916 PMC10654443

[139] Donisi C Pretta A Pusceddu V Ziranu P Lai E Puzzoni M . Immunotherapy and cancer: The multi-omics perspective. Int J Mol Sci. (2024) 25:3563. doi: 10.3390/ijms25063563, PMID: 38542536 PMC10971308

[140] Fritz JV Desai MS Shah P Schneider JG Wilmes P . From meta-omics to causality: experimental models for human microbiome research. Microbiome. (2013) 1:14. doi: 10.1186/2049-2618-1-14, PMID: 24450613 PMC3971605

[141] Cheng S Han Z Dai D Li F Zhang X Lu M . Multi-omics of the gut microbial ecosystem in patients with microsatellite-instability-high gastrointestinal cancer resistant to immunotherapy. Cell Rep Med. (2024) 5:101355. doi: 10.1016/j.xcrm.2023.101355, PMID: 38194971 PMC10829783

[142] Gupta VK Paul S Dutta C . Geography, ethnicity or subsistence-specific variations in human microbiome composition and diversity. Front Microbiol. (2017) 8:1162. doi: 10.3389/fmicb.2017.01162, PMID: 28690602 PMC5481955

[143] Deschasaux M Bouter KE Prodan A Levin E Groen AK Herrema H . Depicting the composition of gut microbiota in a population with varied ethnic origins but shared geography. Nat Med. (2018) 24:1526–31. doi: 10.1038/s41591-018-0160-1, PMID: 30150717

[144] Bolte LA Lee KA Björk JR Leeming ER Campmans-Kuijpers MJE de Haan JJ . Association of a Mediterranean diet with Outcomes for patients treated with immune checkpoint blockade for advanced melanoma. JAMA Oncol. (2023) 9:705–9. doi: 10.1001/jamaoncol.2022.7753, PMID: 36795408 PMC9936383

[145] Merenstein D Guzzi J Sanders ME . More information needed on probiotic supplement product labels. J Gen Intern Med. (2019) 34:2735–7. doi: 10.1007/s11606-019-05077-5, PMID: 31161565 PMC6854121

[146] Mazziotta C Tognon M Martini F Torreggiani E Rotondo JC . Probiotics mechanism of action on immune cells and beneficial effects on human health. Cells. (2023) 12:184. doi: 10.3390/cells12010184, PMID: 36611977 PMC9818925

[147] Suez J Zmora N Zilberman-Schapira G Mor U Dori-Bachash M Bashiardes S . Post-antibiotic gut mucosal microbiome reconstitution is impaired by probiotics and improved by autologous FMT. Cell. (2018) 174:1406–1423.e16. doi: 10.1016/j.cell.2018.08.047, PMID: 30193113

[148] Walter J Maldonado-Gómez MX Martínez I . To engraft or not to engraft: an ecological framework for gut microbiome modulation with live microbes. Curr Opin Biotechnol. (2018) 49:129–39. doi: 10.1016/j.copbio.2017.08.008, PMID: 28866242 PMC5808858

[149] Zmora N Zilberman-Schapira G Suez J Mor U Dori-Bachash M Bashiardes S . Personalized gut mucosal colonization resistance to empiric probiotics is associated with unique host and microbiome features. Cell. (2018) 174:1388–1405.e21. doi: 10.1016/j.cell.2018.08.041, PMID: 30193112

[150] Kristensen NB Bryrup T Allin KH Nielsen T Hansen TH Pedersen O . Alterations in fecal microbiota composition by probiotic supplementation in healthy adults: a systematic review of randomized controlled trials. Genome Med. (2016) 8:52. doi: 10.1186/s13073-016-0300-5, PMID: 27159972 PMC4862129

[151] Fong FLY El-Nezami H Mykkänen O Kirjavainen PV . The effects of single strains and mixtures of probiotic bacteria on immune profile in liver, spleen, and peripheral blood. Front Nutr. (2022) 9:773298. doi: 10.3389/fnut.2022.773298, PMID: 35495948 PMC9039324

[152] Diao X . Antibiotics and proton pump inhibitors suppress the efficacy of immunotherapy against non-small cell lung cancer. Thorac Cancer. (2020) 11:1763–4. doi: 10.1111/1759-7714.13470, PMID: 32374445 PMC7327691

[153] Cortellini A Tucci M Adamo V Stucci LS Russo A Tanda ET . Integrated analysis of concomitant medications and oncological outcomes from PD-1/PD-L1 checkpoint inhibitors in clinical practice. J Immunother Cancer. (2020) 8:e001361. doi: 10.1136/jitc-2020-001361, PMID: 33154150 PMC7646355

